# The chylomicron saga: time to focus on postprandial metabolism

**DOI:** 10.3389/fendo.2023.1322869

**Published:** 2024-01-18

**Authors:** Alejandro Gugliucci

**Affiliations:** Glycation, Oxidation and Disease Laboratory, Department of Research, Touro University California, Vallejo, CA, United States

**Keywords:** chylomicron, triglyceride-rich lipoproteins, lipoprotein lipase, ApoCIII, ANGPTL, MetS, postprandial, atherosclerosis

## Abstract

Since statins have had such tremendous therapeutic success over the last three decades, the field of atherosclerosis has become somewhat LDL-centric, dismissing the relevance of triglycerides (TG), particularly chylomicrons, in atherogenesis. Nonetheless, 50% of patients who take statins are at risk of developing atherosclerotic cardiovascular disease (ASCVD) and are unable to achieve their goal LDL-C levels. This residual risk is mediated, in part by triglyceride rich lipoproteins (TRL) and their remnants. Following his seminal investigation on the subject, Zilversmit proposed that atherosclerosis is a postprandial event in 1979 (1–4). In essence, the concept suggests that remnant cholesterol-rich chylomicron (CM) and very-low density lipoprotein (VLDL) particles play a role in atherogenesis. Given the foregoing, this narrative review addresses the most recent improvements in our understanding of postprandial dyslipidemia. The primary metabolic pathways of chylomicrons are discussed, emphasizing the critical physiological role of lipoprotein lipase and apoCIII, the importance of these particles’ fluxes in the postprandial period, their catabolic rate, the complexities of testing postprandial metabolism, and the role of angiopoietin-like proteins in the partition of CM during the fed cycle. The narrative is rounded out by the dysregulation of postprandial lipid metabolism in insulin resistance states and consequent CVD risk, the clinical evaluation of postprandial dyslipidemia, current research limits, and potential future study directions.

## 1 Introduction

Zilversmit first suggested that atherosclerosis is a postprandial event in 1979, following his groundbreaking studies on the subject (1–4). In essence, the hypothesis states that atherogenesis is mediated in part by cholesterol-rich remnant particles stemming from chylomicrons (CM) and very-low density lipoproteins (VLDL). In spite of the fact that we spend much of the day in the fed state (1, 5–7), analytical difficulties have delayed the progress of a thorough and comprehensive study of postprandial lipid metabolism and determined that most diagnostic and treatment goals only take into account the fasting lipoprotein and lipid levels (8–11).Since statins have had such remarkable therapeutic success over the past three decades, the field has become somewhat LDL-centric and has dismissed the importance of triglycerides (TG) and particularly chylomicrons in atherogenesis (8, 12–14). Nonetheless, numerous individuals using statins still run the risk of developing atherosclerotic cardiovascular disease (ASCVD) and are unable to achieve their target LDL-C levels (15–19). This residual risk, which may reach 50% after receiving effective statin therapy, results from both immunological and lipid abnormalities. The significance of triglyceride rich lipoproteins (TRL) and their remnants in these key lipid abnormalities has gained attention in recent years (8, 16, 20). Ineffective intravascular TG metabolism and recapture lead to the accumulation of circulating TRL and their “remnants” or partially lipolyzed derivatives. Serum TG serve as a proxy for the lipoproteins they are found in, and more especially, for these remnants of chylomicron (and VLDL) catabolism which contain many times as much cholesterol as LDL. It is impossible to overstate the quantitative importance of chylomicrons, which transport between 70 and 90 g of dietary lipids each day, or over 90% of the total (21–25). This fact confirms the necessity for a shift in the field’s focus. The key role of apolipoprotein CIII (apoCIII) as an atherogenic mediator, showcased in the last decade, underscored the crucial role of lipoprotein lipase (LPL) in atherogenesis and led to successful development of antisense oligonucleotides (ASO) and monoclonal antibodies (mAbs). The importance of LPL regulation for the partition of TRL in the fast-fed cycle is also supported by more recent research on angiopoietin-like proteins (ANGPTL) regulators and the ensuing development of ASO (22, 26–30).

Considering the above, this narrative review examines the most recent advances in our knowledge of postprandial dyslipidemia. Discussion topics include the primary metabolic pathways of chylomicrons, highlighting the critical physiological role of lipoprotein lipase and apoCIII, the significance of these particles’ fluxes in the postprandial period, their catabolic rate, the complexity of testing postprandial metabolism and the role of angiopoietin-like proteins in the partition of CM during the fed cycle. The dysregulation of postprandial lipid metabolism in insulin resistant states and subsequent CVD risk, the clinical evaluation of postprandial dyslipidemia, present research constraints, and potential subsequent study directions round up the narrative.

## 2 Chylomicrons

### 2.1 Chylomicron structure and function

**Figure 1:**
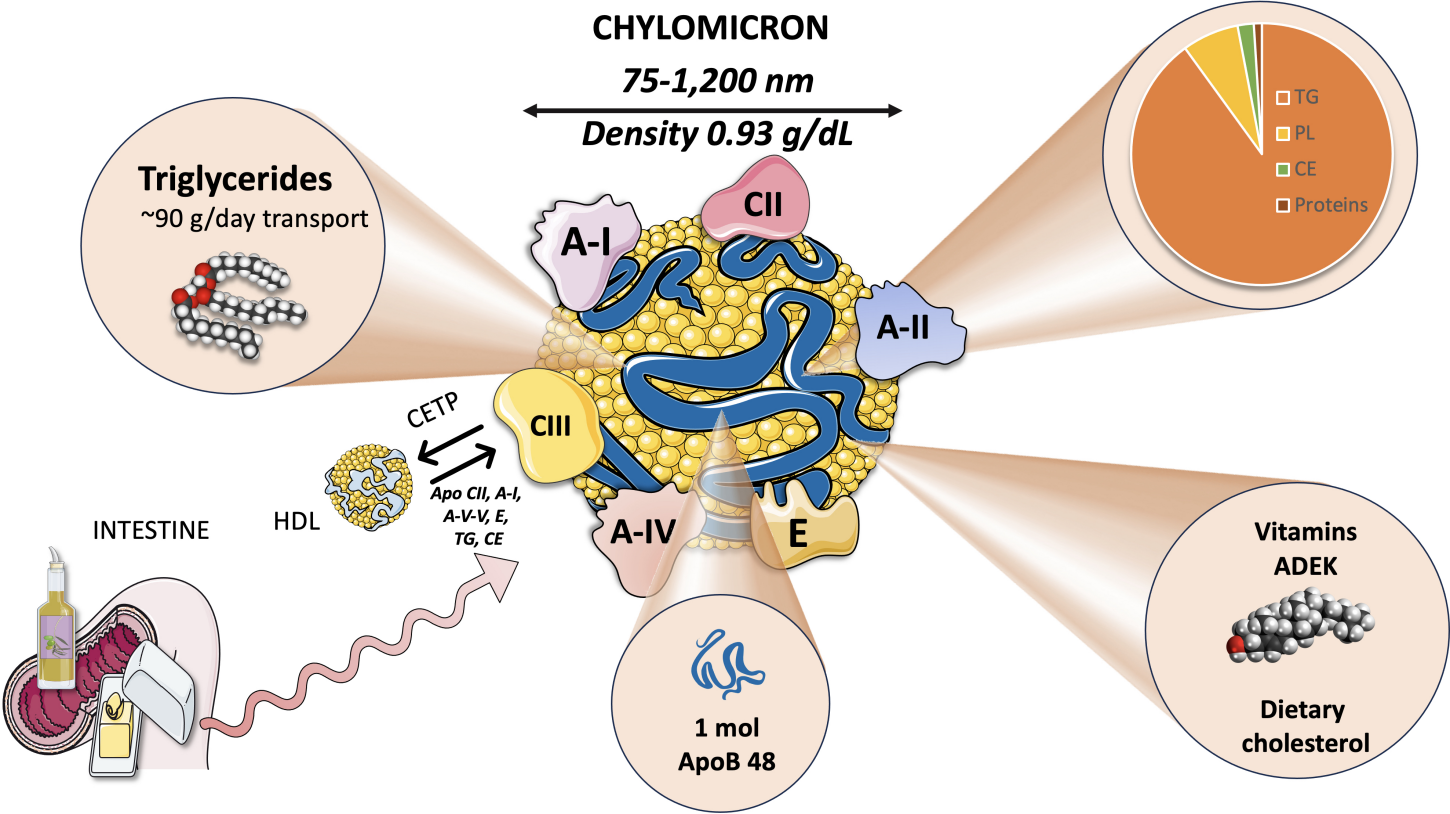
Chylomicrons: structure and function key properties.

Chylomicrons are large triglyceride-rich lipoproteins produced by enterocytes from dietary lipids, specifically cholesterol and fatty acids. While TG make up most of the lipid core of chylomicrons, they also transport phospholipids and esterified cholesterol, just like other lipoproteins (**Figure 1**). CM are the largest lipoproteins, ranging from 75-1,200 nm in diameter, the largest chylomicrons are indeed visible in the optical microscope. CM have a density below 0.93 g/ml and they have the lowest protein composition of any lipoprotein at just 2%, which explains why they have such a low density during ultracentrifugation. Actually, they float in plasma left overnight in the refrigerator, a classical and simple test to determine their presence. Of note, all liposoluble vitamins in the diet are also carried by chylomicrons (8, 31–33).

#### 2.1.1 Chylomicron proteins

##### 2.1.1.1 Intrinsic

The primary non*-exchangeable protein*, apolipoprotein B48 (a spliced product of the apoB100 gene), is the backbone structural protein. A single apoB48 protein is present in each chylomicron particle. Microsomal triglyceride transfer protein (MTTP) mediates the lipidation of apoB48 in enterocytes as shown in Section 3. Other apolipoproteins, such as ApoAI, AII, AV, CII, CIII, and ApoE, are present (33–35).

##### 2.1.1.2 Exchangeable proteins

The metabolism of CM is greatly influenced by lipoprotein lipase (LPL) and its modulators. Several surface chylomicron proteins are critical for their metabolism and reflect crucial interaction with HDL as discussed in Section 4. LPL activity is stimulated by apoCII and suppressed by apoC-III. Familial hyperchylomicronemia may be produced (among others) by mutations in the protein ApoAV, which is involved in the activation of LPL (Section 4) and increasing TG lipolysis. Another HDL-derived protein that mediates the liver uptake chylomicron remnants is apolipoprotein E (14, 36)Intestinal apoAI carried first by nascent CM represents 30% of HDL apoAI.

## 3 Chylomicron production

### 3.1 Digestion and absorption of fats

**Figure 2:**
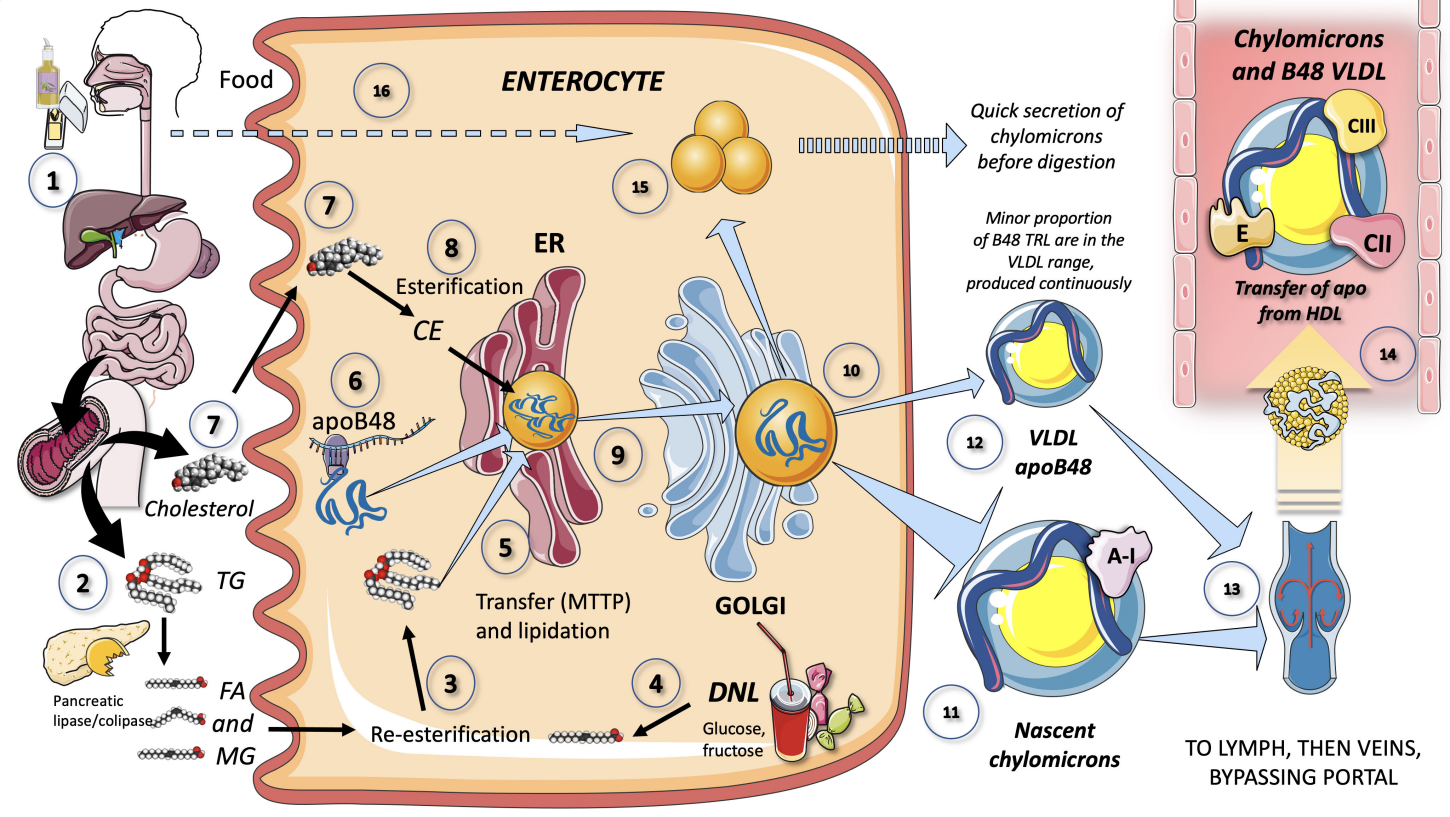
Production of chylomicrons and VLDL apoB48 by the enterocyte (1). Lipids in the diet are (2) digested by pancreatic lipase, which is activated by liver co-lipase, in a complex system of micelles, resulting in the absorption of fatty acids and monoglycerides (3). Fatty acids are re-esterified by the enterocyte and the resulting TG serve to lipidate apoB48 (4). Another source of FA may be those produced by *de novo* lipogenesis (DNL) driven by excess fructose. TG are transported by MTTP in their movement across the ER and Golgi apparatus. apoB48 (6) is a truncated, spliced form of apoB100, which lacks the LDL receptor binding sites. Cholesterol from the diet (7) is absorbed via the Niemann-Pick receptor and is also esterified (8) before integrating chylomicrons (9). Much less is known about the transit and steps in chylomicron production as compared to VLDL. The resultant CM (10), which are very large particles, are (11) secreted into the circulation as nascent CM containing also apoAI. It has recently been shown that some of the apoB48-containing lipoproteins coming from the intestines are also in the VLDL size range (12). CM (and VLDL-ApoB48) secreted into the circulation contain phospholipids, cholesterol esters, and the liposoluble vitamins and are first transported by the lymph (13), reaching the venous, and finally the arterial circulation in a third step (14). CM receive peripheral proteins apoCII and CIII, E and others from HDL in the circulation. Some TG are stored as an intracellular pool of TG droplets (15) that can be called upon very quickly when the next meal comes, following signals stemming from the intake of more food and even before it reaches the intestines (16).

Because TG are nonpolar, they cannot be transported from the gut lumen directly into intestinal epithelial cells through the diet. Instead, pancreatic and stomach lipases hydrolyze dietary TG to produce free fatty acids (FA) and 2-monoglycerides (2-MG), which are then transported into intestinal epithelial cells. It is thought that passive diffusion and a saturable protein-mediated mechanism both contribute to the uptake of FA and 2-MG by intestinal epithelial cells. As depicted in **Figure 2**, pancreatic lipase breaks down dietary fat with the help of liver co-lipase in a sophisticated system of micelles that eventually results in the generation of FA and 2-MG. Concurrently, FA (14, 32, 36) and lysophospholipids are produced by the hydrolysis of phospholipids, which is mostly accomplished by pancreatic phospholipase A2. Cholesterol is found in the intestinal lumen as both non-esterified cholesterol and cholesteryl esters and is derived from the diet as well as the liver via bile, its only mechanism of excretion. The intestine can only absorb non-esterified cholesterol (**Figure 2**). Cholesteryl esters are hydrolyzed in the intestinal lumen to form non-esterified cholesterol and FA. About half of the cholesterol ingested in the gut is eliminated in the feces. The Niemann-Pick C1-like 1 (NPC1L1) protein is primarily responsible for intestinal cholesterol absorption, which is blocked by ezetimibe (a cholesterol-lowering drug). NPC1L1 is also involved in the absorption of plant sterols. Passive diffusion mediates the absorption of lysophospholipids (8, 33, 36).

### 3.2 Production of chylomicron by enterocytes

**Figure 2** provides a simplified overview of the key characteristics of enterocyte-mediated chylomicron production. The chylomicron assembly pathway, which transports all dietary fat (more than 70 g per day) except for short chain fatty acids, is far less well understood than the liver VLDL assembly pathways. Many hypotheses have been proposed to explain the formation of these massive TRL. In a nutshell, after being absorbed by the enterocyte, fatty acids and 2-MG are re-esterified to form TG, which is the initial point of control for the composition of these molecules, i.e., the proportion of saturated fatty acids to mono, poly, and saturated fatty acids. apoB48, a shortened, spliced version of apoB100 that lacks the LDL receptor binding region, serves as the chylomicron’s backbone (33, 34, 37–39). When apoB48 passes through the ER and Golgi apparatus, it is lipidated with TG, phospholipids, and cholesterol in a similar, but less well-described mechanism than that of liver VLDL (**Figure 2**).

#### 3.2.1 Re-esterification

Non-esterified cholesterol, FA, 2-MG, and lysophospholipids are transported to the endoplasmic reticulum (ER) in enterocytes. ACAT (acyl-coA:cholesterol acyltransferase) esterifies non-esterified cholesterol in the ER. Two transporters found in the brush border, ATP-binding cassette G5 (ABCG5) and ATP-binding cassette G8 (ABCG8), reject non-esterified cholesterol from the cytosol back to the intestinal lumen. ABCG5 and ABCG8 are also in charge of transferring plant sterols back to the lumen, reducing plant sterol buildup in the body. Specific fatty acid-binding proteins (FABPs) transport absorbed FA and 2-MG to the ER. In the ER, membrane-bound 2-MG acyltransferases (MGATs) esterify 2-monoacylglycerol with FA, resulting in the production of diacylglycerol, which is subsequently converted to triglycerides by the action of diacylglycerol acyltransferases (DGAT). Furthermore, diacylglycerol is coupled with choline by choline-transferase and with ethanolamine by ethanolamine-transferase for the formation of phospholipids in the ER (40).

##### 3.2.1.1 Pathway of the MGAT (monoacylglycerol acyltransferase), the main mechanism for TG production in the small intestine

The MGAT pathway contributes to most of the TG synthesis in the small intestine and is crucial for the absorption of dietary fats. It involves the microsomal enzyme MGAT, which catalyzes the acyl-CoA-dependent esterification of 2-MG to produce 1,2-DG, a precursor for TG biosynthesis. About half of the TG found in CM released from the small intestine is created by the lipolysis and re-esterification of TG found in enterocytes’ lipid droplets. Of note, in humans, lipids from a single meal have been found in the CM formed after a second meal (41–43).

##### 3.2.1.2 TG production and diacylglycerol acyltransferases, the role of trioses and glyceroneogenesis

The last process of TG biosynthesis, which is mediated by DGAT, can use the 1,2-DG produced by either the MGAT or glycerol-3-P (G3P) pathways as a substrate. The latter is the pathway that uses glycolysis trioses to yield G3P as a backbone for newly synthesized TG, also known as glyceroneogenesis. At the ER, DGAT catalyzes the formation of an ester bond between the free hydroxyl group of the sn-3 carbon of 1,2-DG and the carboxyl group of a fatty acid (from a fatty acyl-CoA), resulting in TG. TG can be directed to the ER lumen where it can be processed as discussed below (40, 42).

It is generally accepted that the main mechanism for TG production in the small intestine, where it plays a significant role in dietary fat absorption, is the MGAT pathway. Many of the iso-enzymes involved in the synthesis of intestinal lipids have now been discovered, but there are still many unsolved problems. The development of small molecule inhibitors that target the enzymes in the small intestine that produce TG would be substantially aided by structural knowledge and is the focus of current research.

#### 3.2.2 Apo B48, a truncated product of the apoB100 gene (VLDL and LDL)

As shown in **Figure 2**, apolipoprotein (apo) B48, which is only found in the intestine, encounters TG in the ER via the action of microsomal triglyceride transfer protein as well as cholesterol, phospholipids, and ApoAIV, to produce pre-chylomicrons (40). The ubiquitin-proteasome system rapidly degrades apoB48 that is not bound to lipids. Pre-chylomicrons are synthesized in the ER and transported to the cis-Golgi by pre-chylomicron transport vesicles. ApoAI associates with pre-chylomicrons in the Golgi to create mature chylomicrons, each bearing a single molecule of apoB48. Exocytosis then causes CM to be discharged from the basolateral side of the enterocyte and migrate through the lamina propria before accessing the lymphatic lacteals (8, 31, 33, 34, 44). Cytosolic droplets associated with certain proteins, such as perilipins, can contain ER lipids that have not been used to form chylomicron (44). A noteworthy finding is that, in contrast to what was previously assumed, new research has shown that some TG are retained and stored as cytosolic droplets (**Figure 2**) in the intestines and that the first source of TG produced after a meal may derive from past food intake (45). According to this view, intestinal cells do not “have to wait” for dietary lipids to cross the enterocyte border to begin secreting stored TG in CM after a meal has been consumed (8, 21, 24, 46, 47). The production of chylomicrons has been connected to the taste-gut-brain axis, which may help to explain why fat or glucose simply needs to be tasted and not necessarily ingested to generate CM (48–50).

Primordial, lipid-poor, apoB48-containing lipoproteins and large CM may assemble through different processes. Nascent CM (CM that have just been secreted), as seen in **Figure 2**, are released into lymphatic channels and go to the thoracic duct where they are delivered to the left subclavian vein along with their cargo of dietary lipids and fat-soluble vitamins. Most dietary lipids therefore do not pass via the hepatic portal system like other nutrients do. Only short and medium-chain fatty acids, which comprise 2-4 (acetic, propionic and butyric acid) and 6–12 carbon atoms respectively, are directly absorbed into the portal circulation (8, 47, 51, 52).

#### 3.2.3 ApoB48-VLDL

Recent research has shown that TRL that include apoB48 also exist and stem from two sources. First, it has been shown that CM remnants that enter the liver can be utilized again to rebuild VLDL (53). Specifically, research demonstrates that the LDL receptor and the LDL-receptor-related protein (LRP) facilitate the seamless integration in VLDL of some apoB48 in CM remnants following fat consumption. The mechanism is either identical to or comparable to the hepatic process of forming VLDL apoB100 (54). Secondly, research also has shown that apoB48-VLDL are released both continuously and at a low rate during a fast and at a higher rate following a meal (**Figure 2**). Following a fat-rich meal, apoB48 appeared in the chylomicron, VLDL_1_ and VLDL_2_ fractions (21, 54). ApoB48-containing particles were secreted directly into both the chylomicron and VLDL fractions. During fat absorption, whilst most triglyceride entered the circulation in chylomicrons, the majority of apoB48 particles were secreted into the VLDL density range (21, 54).

#### 3.2.4 Intestinal *de novo* lipogenesis

The metabolic mechanism that serves to synthetize fatty acids from excess carbohydrates is known as *de novo* lipogenesis (DNL). These fatty acids can then be converted into triglycerides (TG), which are used to store energy (37, 55). DNL occurs mostly in the liver and adipose tissue under normal settings and was previously thought to be a small contributor to serum TG homeostasis. However, recent research suggests that intestinal DNL may play a role in serum lipid concentration in people who eat a high carbohydrate diet (8, 55–59). DNL drives the movement of carbons from glucose to fatty acids through a coordinated set of enzyme processes. The initial reaction in this chain is the conversion of citrate (an exporter of mitochondrial acetyl-CoA) to acetyl-CoA by ATP-citrate lyase. Acetyl-CoA is then carboxylated to malonyl-CoA by acetyl-CoA carboxylase. Malonyl-CoA acts as a substrate for FA synthesis as well as an allosteric inhibitor of FA transport to the mitochondrion. This process partitions most newly synthesized FA to TG synthesis. The primary rate-limiting enzyme in the conversion of malonyl-CoA to palmitate is fatty acid synthase (FASN) which employs large amounts of reduction equivalents in the form of NADPH from the pentose cycle and malic enzyme. Its main end-product, palmitate, is subsequently transformed into other fatty acids through a variety of desaturation and/or elongation reactions. In addition DNL also produces minor quantities of stearate and shorter fatty acids. The role of fructose in DNL is well established in the liver and recent research strongly suggests it also occurs in the intestine (57, 60).

##### 3.2.4.1 The role of diabetes in intestinal *de novo* lipogenesis

Numerous studies show that type 2 diabetes is associated with an increase in DNL in enterocytes. Enterocytes isolated from insulin-resistant fructose-fed hamsters have considerably higher intracellular levels of non-esterified cholesterol, esterified cholesterol, and triglycerides, which may indicate enhanced lipogenesis (61).

In animal models of diabetes, increased expression of MGAT, DGAT, and the mature form of sterol regulatory element binding transcription factor 1c (SREBP-1c), a transcription factor promoting lipogenesis, has also been observed in intestinal cells. According to recent studies on humans (62–64), obese subjects with insulin resistance had higher levels of SREBP-1c, apoAIV, and fatty acid binding protein (FABP) expression in their intestines. This suggests that increased fatty acid availability in insulin-resistant conditions may also contribute to the excess CM formation (49, 50, 62–64).

As a result, increased CM synthesis by the intestine is a significant feature not only in people with type 2 diabetes but also in insulin-resistant people who are not diabetic, suggesting that insulin resistance is likely to be the primary mechanism implicated in its pathogenesis (19, 61). Hyperglycemia itself may exacerbate chylomicron overproduction in type 2 diabetes. Duodenal co-infusion of glucose with Intralipid or fructose with Intralipid significantly increases the synthesis of apoB48 in healthy males. Additionally, healthy males with hyperglycemia brought on by glucose infusion produce higher apoB48-containing lipoproteins.

### 3.3 Regulation of chylomicron production and secretion

#### 3.3.1 Insulin has a key role

Insulin has a substantial impact on intestinal lipid metabolism both directly and indirectly by inhibiting the release of FA from adipose tissue. Regarding the elements that influence the synthesis of CM in the liver and gut, there is a parallelism. Chylomicron production and release are in fact regulated by both FA and insulin. In individuals with insulin sensitivity, insulin reduces the release of apoB48-containing lipoproteins from the intestine. However, in individuals with insulin-resistant diseases, such as metabolic syndrome (MetS) or type 2 diabetes, this regulatory function is weakened, resulting in an excess of CM and apoB48-VLDL (65–68).

Preclinical research suggests that insulin decreases the formation of chylomicron and the secretion of apoB48 in cultured human fetal jejunal explants (65). Forkhead box protein O1 (FoxO1) suppression by insulin partially mediates the inhibitory action of insulin on the expression of the MTTP gene. Furthermore, evidence points to insulin as a promoter of post-ER, presecretory proteolysis of apoB48 (65)After a lipid-rich meal, plasma apoB48 significantly decreased after a euglycemic-hyperinsulinemic clamp in comparison to no insulin infusion (66, 69). When insulin (+glucose) was infused into healthy men, a reduction in apoB48 -containing lipoprotein synthesis of 50–52% compared to saline infusion, and a reduction of 16–21% when the insulin-induced lowering of FA was avoided by concurrent intralipid infusion. These findings imply that insulin has a direct effect, in addition to lowering plasma FA levels, in reducing the formation of intestinal lipoproteins (65, 66, 70).

#### 3.3.2 Carbohydrates

Meals high in monosaccharides can raise postprandial triglyceride levels. Isocaloric substitution of monosaccharides into mixed meals, on the other hand, has no discernible stimulatory or inhibitory effect on postprandial triglycerides. Mechanistic investigations with carbohydrate consumption or plasma glucose elevation have shown increased CM production (34). The mechanisms underlying this regulation remain largely unclear, however they may involve increased intestinal DNL and mobilization of lipids stored in the intestine (8, 59, 71). Our recent findings that fructose restriction reduces apoB48 and remnants supports this contention (72).

The role of incretins in intestinal lipid absorption and metabolism is discussed in Section 7.

## 4 Chylomicron intravascular catabolism

### 4.1 Overview

Strict control over triglyceride transport is necessary for survival. When FA are needed for energy production, they are released from TG in fat depots and directed in the right direction. Conversely, TG are conserved for usage when they are abundant. LPL (lipoprotein lipase), HL (hepatic triglyceride lipase), and EL (endothelial lipase) are a family of enzymes that operate to remove TG from VLDL and chylomicrons (21, 46, 47, 73). While HL and EL favor smaller VLDL particles, remnants, IDL (intermediate-density lipoproteins), and HDL, LPL is mostly active in CM and larger VLDL. A complete loss of LPL causes fasting hyperchylomicronemia with severe hypertriglyceridemia. Between 50% and 70% of the core triglyceride is normally eliminated by lipolysis of CM in non-hepatic tissues, and larger CM are broken down into smaller chylomicron remnant particles (74).

### 4.2 Lipoprotein lipase

**Figure 3:**
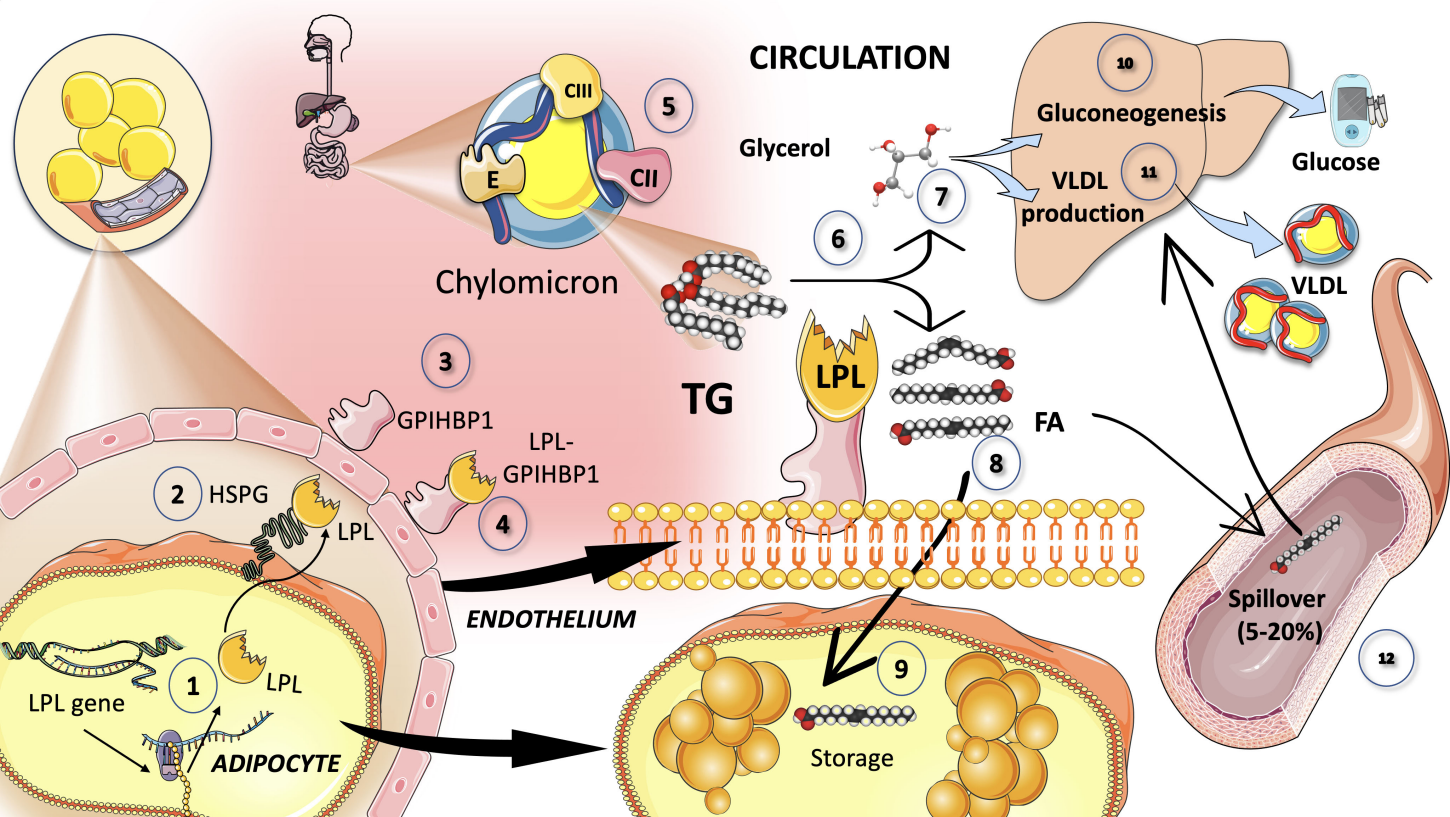
Catabolism of chylomicron: Lipoprotein lipase intravascular TG hydrolysis (1). Lipoprotein lipase is synthesized by the cells in oxidative tissues such as muscle (skeletal and myocardium) as well as adipocytes and the mammary gland, among others (2). It is a very complex enzyme that needs heparan sulfate proteoglycans (HSPG) on the surface of the cells to assist in the transcytosis of the molecule to the luminal face of the capillaries (3). Glycosylphosphatidylinositol anchored high-density lipoprotein binding protein 1 (GPIHBP1) serves to anchor LPL on the luminal surface of the endothelial cells and supports the conformation of LPL to an active lipolytic enzyme (4). LPL acts on CM (5) to hydrolyze TG (6) into glycerol (7) and free fatty acids (8). Free fatty acids are employed for storage in adipocytes, as shown in (9). Glycerol is taken up by the liver to fuel gluconeogenesis in fasting (10) but mainly TG synthesis in the fed state (11). Some of the fatty acids (that may amount to 5 to 30%) remain in the circulation and are referred to as *spill over* fatty acids (12) which are taken up by the liver to be repackaged as TG, secreted as VLDL. LPL has a very complex regulation: its main activators are insulin, apoCII, AIV, and AV and its main inhibitors are apoCIII and angiopoietin-like protein (ANGPTL) 3, 4, and 8.

Lipoprotein lipase, which catalyzes the release of FA for storage by adipose tissue or oxidation as an energy source by muscle, is the main enzyme in the intravascular breakdown of TG in circulating TRL (**Figure 3**). LPL is not produced by the liver, but rather by macrophages, adipose tissue, skeletal and cardiac muscle, and the brain. LPL operates intravascularly, precisely at the endothelium’s luminal surface. A variety of modulators acting in concert control the intricate regulation of LPL production and activity (74–78). For instance, insulin seems to regulate post-transcriptional and post-translational levels of LPL activity in adipocytes (**Figure 3**).

As shown in **Figure 3**, LPL is produced and matured in the ER before being transported by cells (such as adipocytes, skeletal or cardiac myocytes, etc.) to the endothelial surface of capillaries, where it is activated. In processing immature LPL, the ER is essential, and numerous significant regulators are involved. LPL is assisted in its transcytosis to the luminal face of the capillaries by binding to heparan sulfate proteoglycans (HSPG) on the cell surface. LPL is guided from its site of synthesis to the endothelial surface by the proteins lipase maturation factor 1 (LMF1) and glycosylphosphatidylinositol­anchored HDL­binding protein 1 (GPIHBP1), which also aid in anchoring LPL to endothelial cells in capillaries. Additional investigation indicates that LPL can also be active when it forms a complex with GPIHBP1, contrary to earlier findings that showed it could only be active as a homodimer (39, 79–82).

#### 4.2.1 Main regulators of LPL

The rapid adaptive alterations of LPL to dietary changes are governed by insulin responses, together with the moderating effects of other hormones and proteins that control necessary variations in the lipolytic rates between fasting and postprandial states (**Figure 3**). Numerous apolipoproteins present on the surface of TRL have various effects on their metabolism. ApoB (48 or 100) is not transported between TRL and HDL particles, whereas apoCI, apoCII, apoCIII, and apoE are, depending on the nutritional or metabolic state. LPL activity is increased by apoCII and apoAV while decreased by apoCI, apoCIII, and apoE (83). The rate-limiting element in lipolysis, which is mediated by LPL, appears to be apoCII. A dual apoCII mimetic-apoCIII antagonist peptide’s ability to reduce plasma triglyceride levels was discovered because of this research (84, 85)These agents are currently in the early stages of development. Cross regulation of LPL among tissues during the fast fed cycle is discussed further below (21, 46, 86).

### 4.3 Interaction of CM with HDL

**Figure 4:**
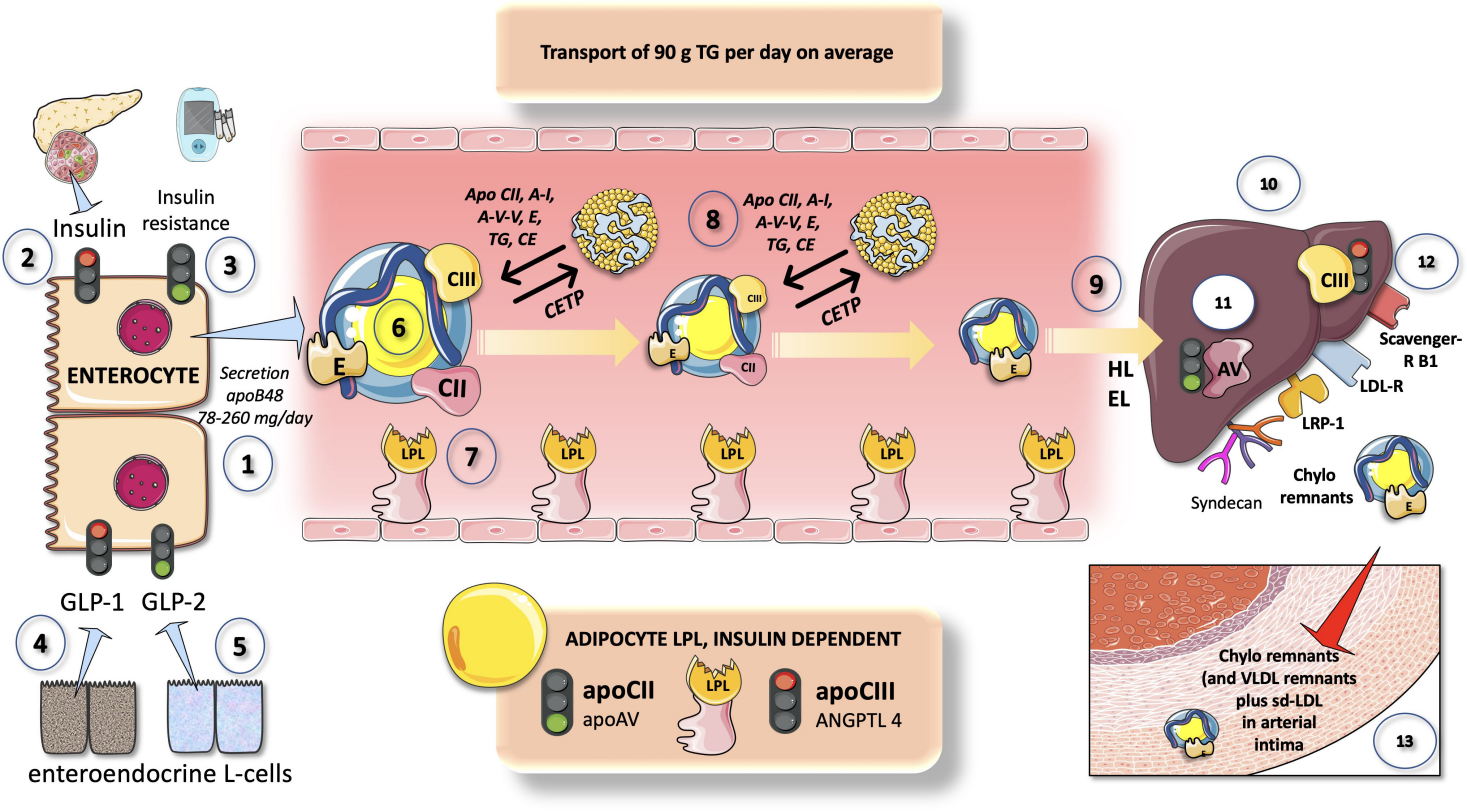
Overview of chylomicron metabolism and its regulation. CM are secreted by enterocytes at a rate of 78-260 2-MG apoB48 per day (1) that transports about 90 g of dietary TG per day (on a regular diet). Insulin (2) is a negative modulator of chylomicron secretion, as a corollary states of insulin resistance promote chylomicron production (3) Different entero-endocrine L cells secrete GLP1 (4) which inhibits and GLP2 (5) which increases chylomicron production. CM in the circulation (6) are acted upon by LPL as shown in **Figure 3**. CM exchange peripheral proteins and lipids with HDL which is a key support of their intravascular metabolism. They acquire apoCII (main activator) and apoCIII (main inhibitor) of LPL as well as apoE which is crucial for liver reuptake of remnants. In turn HDL gets some of its apoAI from them. With the concourse of CETP surface phospholipids, TG and core cholesterol esters are exchanged with HDL. In minutes CM drastically lose their TG and surface PL and are acted upon by HL and EL (9) to become cholesterol-rich remnants. These are taken up by the liver (10) through at least four different mechanisms as depicted: LDL receptor, LDL receptor like-protein1, scavenger receptor B1 and syndecan 1. This is the pathway by which dietary liposoluble vitamins and cholesterol finally reach the liver. Uptake is stimulated by apoAV (11) and inhibited by apoCIII (12). Delays in any part of this process result in the transfer of the remnants into the arterial intima (13). Remnants are less abundant but contain several times more cholesterol esters as LDL and are equally or more atherogenic.

Nascent chylomicron (and VLDL particles from the liver), respectively, enter plasma and interact with HDL from which they get exchangeable surface apolipoproteins (**Figure 4**).

These include apoCII, which activates LPL, apoAV, and others, most notably apoCIII and apoCI, which can hinder it. Additional proteins that can be derived from HDL include apoE, apoAI, and apoAII. Following the onset of lipolysis, as depicted in **Figure 4**, some exchangeable apos are shed back to HDL, and phospholipid and cholesteryl ester transfer protein (CETP), respectively, mediate this exchange of surface and core lipids with HDL and LDL (87, 88).

Since CETP transfers TG to the HDL and cholesterol to the remnant or LDL particle, this process explains why hypertriglyceridemia and low HDL cholesterol levels are frequently associated. Due to this, low HDL cholesterol is not harmful in and of itself, but rather a sign for TRL dyslipidemia (8, 89). It serves as a stand-in for an indicator of the existence of atherogenic remnants in the circulation. It is impossible to overstate the importance of HDL in these processes, although this review will not go into great depth about it. Reviews on the subject are recommended to the reader (89–91).

### 4.4 ApoCIII is a potent inhibitor of LPL

**Figure 5:**
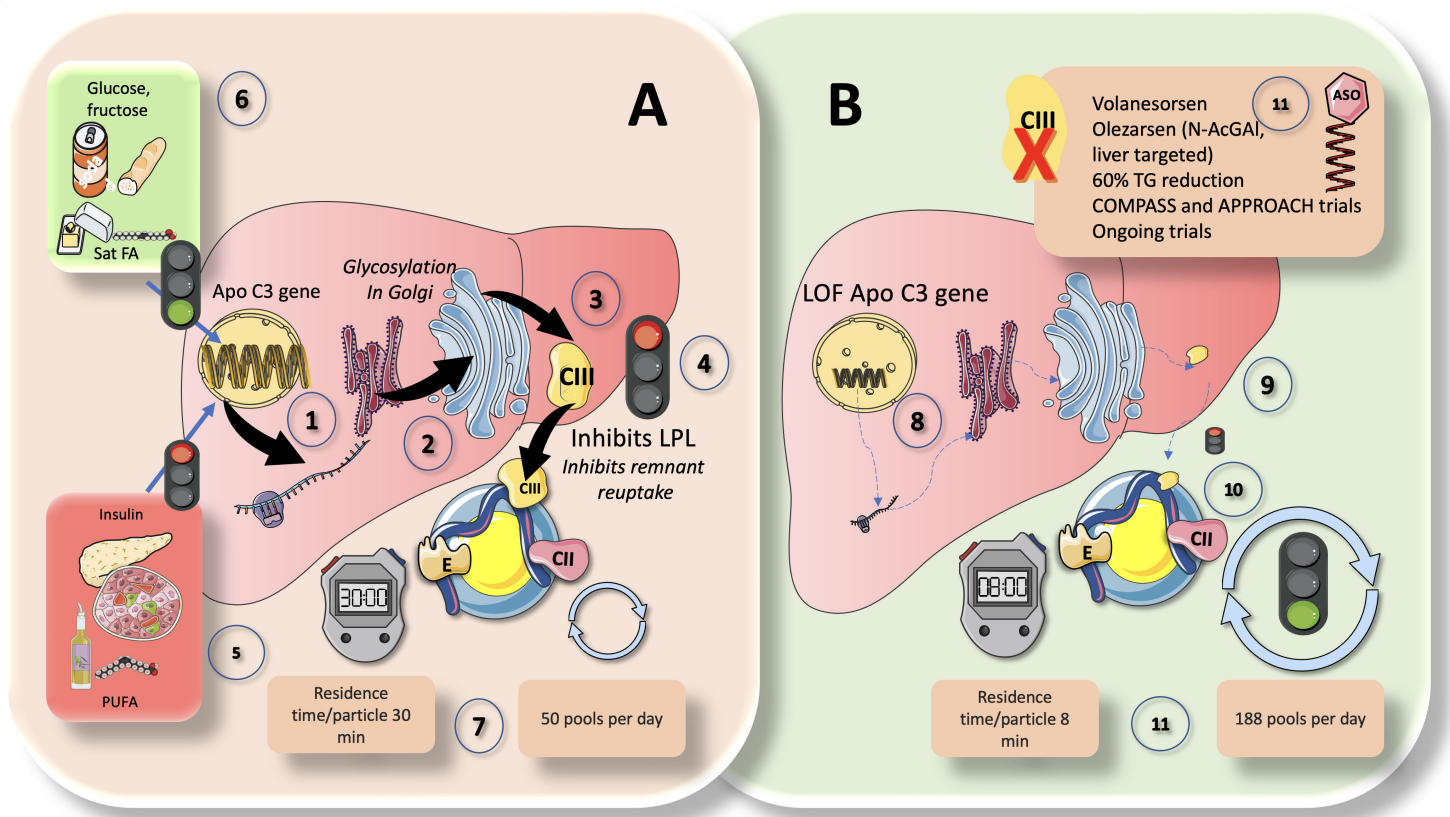
ApoCIII, a crucial inhibitor of LPL activity: physiological and pharmacological standing. Comparison of **(A)** chylomicron fluxes in healthy control subjects vs **(B)** healthy subjects with loss-of function apoCIII (1). Hepatocytes have the apoCIII gene for a protein of about 8 kilodaltons that gets glycosylated in the Golgi apparatus (2) and circulates in three isoforms with 0, 1 (most abundant), or 2 sialic acid residues (3). ApoCIII in CM (which get it by transfer from VLDL or from HDL) potently inhibits lipoprotein lipase activity (4) and acts as a counterpart of the main activator, which is ApoCII. Other key inhibitors are ANGPTL3-4 and 8, as we show later. ApoCIII regulation is complex but can be summarized as follows (5): The two main inhibitors are insulin and polyunsaturated fatty acids, whereas (6) the main enhancers are glucose, fructose, and saturated fatty acids. In this regard, insulin resistance (IR) negates the inhibition promoting increased expression of an ApoCIII delayed catabolism of TRLs, as seen in metabolic syndrome and diabetes, and at the same time produces hyperglycemia, which directly stimulates the production. Diets very rich in sugar or saturated fat enhance the production of apo CIII. Excess activity of apoCIII result in increased residence time of VLDL and chylomicron remnants. State-of-the art stable isotopes studies show that, as depicted in **(A)** (7), that average chylomicron particle residence time is 30 minutes at usual levels of apoCIII and turnover is 50 pools per day. In **(B)**, we illustrate changes found in humans with LOF mutations of apoCIII. Defective gene (8) leads to poorly or inactive protein (9) in the surface of CM not inhibiting LPL (10). This results in a dramatic increase in turnover, with more than threefold reduction in residence time and concurrent increase in turnover from 50 to 188 pools per day (11). The key role of apoCIII in this process as well as results from animal and human loss of function studies have uncovered the potential role of apoCIII inhibitors (ASO) as a therapeutic avenue for hypertriglyceridemia (11), as we further discuss in this review.

ApoCIII is an essential regulator of plasma TRL levels and has been directly related to an elevated risk of ASCVD. LPL activity is inhibited by ApoCIII, although LPL-independent mechanisms also seem to play a role in its proatherogenic effects. Hepatocytes, as seen in **Figure 5A**, express the ApoCIII gene, which codes for a protein of roughly 8 kDa. ApoCIII circulates in three isoforms with 0, 1, or 2 sialic acid residues after being glycosylated in the Golgi apparatus (71, 79, 80, 92). The distribution of isoforms may influence the final activity. Its control is intricate, but it can be summed up as follows and illustrated in **Figure 5**. Insulin and polyunsaturated fatty acids are the two primary inhibitors, while glucose, fructose, and saturated fatty acids are the main enhancers. Effects that stimulate glucose are mediated by Carbohydrate response element binding protein (ChREBP) and Hepatocyte Nuclear Factor 4 (HNF4). Studies conducted both *in vivo* and *in vitro* have demonstrated that insulin blocks the forkhead box protein O1’s ability to produce ApoCIII (82, 93, 94).

The combined effects of insulin and glucose appear to be the logical cause of the dysregulation of apoCIII metabolism seen in diabetics. Regarding this, insulin resistance both generates hyperglycemia, which directly promotes production, and negates the inhibition by promoting higher expression of an ApoCIII delayed catabolism of TRL as demonstrated in MetS and diabetes (**Figure 5**). Diets high in sugar or saturated fat increase apoCIII synthesis (21, 24, 46). Additionally, the expression of ApoCIII is stimulated by saturated FA and inhibited by polyunsaturated FA (PUFA).

In chylomicrons and VLDL, ApoCIII is a powerful inhibitor of LPL activity and serves as a counteracting factor to ApoCII, which is the primary activator. VLDL and chylomicron remnants spend more time in the circulation when apoCIII is overactive. Recent elegant kinetic research on loss-of-functions (LOF) apoCIII in humans has provided striking proof (39). As illustrated in **Figure 5A**, in controls, CM residence time per particle was 30 min, and turnover was 50 pools per day. LOF subjects (**Figure 5B**), conversely displayed an almost 4 times faster metabolism: CM residence time per particle was only 8 min, and turnover was 188 pools per day. The important role that apoCIII plays as an LPL inhibitor as well as the findings from studies on animal and human loss of function have also shown the potential use of apoCIII inhibitors as a treatment approach for hypertriglyceridemia (27, 30, 95). As we previously demonstrated, apoCIII is elevated even in adolescents if they have obesity or simply IR; dietary intervention by reducing fructose consumption is a powerful reducer of apoCIII (59, 72, 96–99). Additionally, ApoCIII blocks remnant reuptake, particularly in rodents (92).

### 4.5 ApoAV is a potent promoter of CM (and VLDL) lipolysis

The liver is the primary site of synthesis for the unique lipid-modulating protein known as apoAV, which also affects the gut, blood flow, liver, and adipose tissue (74, 100, 101). The apolipoprotein gene cluster APOA1/C3/A4 on human chromosome 11q23 is known to influence lipid metabolism. ApoAV has been referred to as “a potent TG reducer” and has been described as having a “low concentration, high impact” since its discovery. When measured on a molar basis, it has a plasma concentration in humans that is incredibly low relative to other apolipoproteins, ranging from 20 to 500 ng/ml, which is roughly 1,000 times lower than apoB and 10,000 times lower than apoAI (74, 100).

According to some publications, ApoAV may be recycled by enterohepatic circulation and behaves more like an incretin hormone. Furthermore, the synthesis and secretion of CM in the small intestine may be significantly impacted by low amounts of apoAV secreted into bile. A remarkable trait of apoAV is its ability to escape luminal proteolysis and be taken up intact by enterocytes. Numerous studies support the notion that apoAV contributes significantly to the enhancement of plasma lipoprotein clearance via enhancement of TRL lipolysis and enhancement of liver absorption of remnant particles (100). Will it develop into a drug target?

## 5 Chylomicron liver reuptake

During passage through the hepatic sinusoids, remnant particles are further hydrolyzed by hepatic triglyceride lipase (HL) and gain additional apoE, which makes it possible for them to bind to and be taken up by proteins on the surface of liver cells, such as the LDL receptor (LDLR), LDL-like receptor protein-1 (LRP-1), and the heparin sulfate proteoglycan syndecan-1 (**Figure 4**). Additionally, as will be discussed later, their apoAV content promotes receptor-mediated remnant absorption (31, 62). Studies on humans show that apoCIII plays a significant role as an inhibitor of LPL-mediated lipolysis, whereas studies on mice show that apoCIII plays a major role as an inhibitor of the liver’s ability to remove remnants of VLDL and chylomicrons. Indeed, in humans, the LOF of apoCIII has little impact on the liver’s ability to remove remnants, unless LPL activity is drastically diminished or nonexistent (39).

## 6 Postprandial lipoprotein metabolism

The wave of TRL that appears in the post-prandial state is made up of CM and VLDL carrying apoB48. These TRL represent a dynamic ‘load’ in addition to VLDL that is released almost continuously throughout the day.

### 6.1 An apparent paradox, the larger increases in postprandial TG belong to VLDL

**Figure 6:**
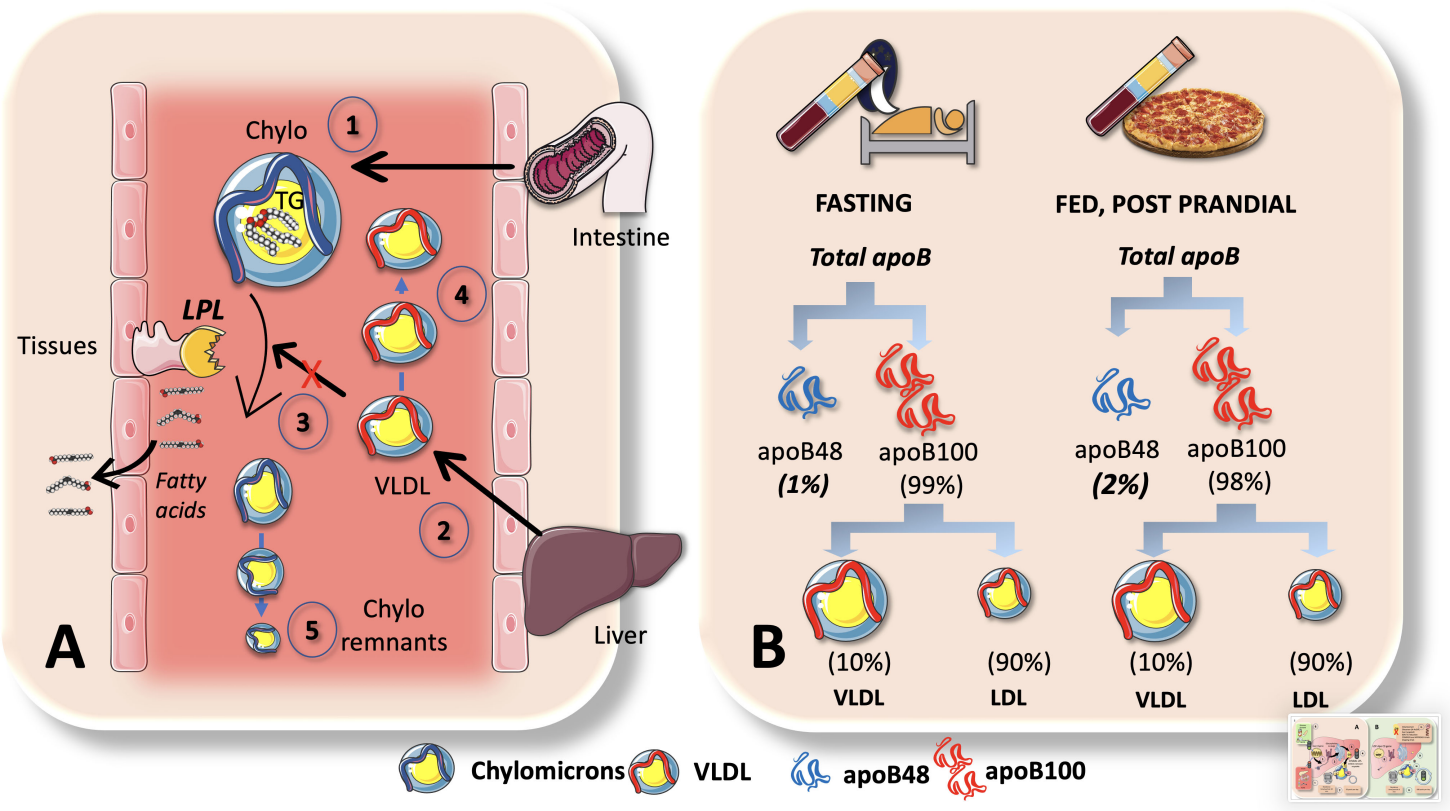
Chylomicrons are preferential substrates for LPL: consequences in the postprandial period. **(A)** In the postprandial period there is not only a surge of chylomicrons (1) in plasma but of VLDL (2) as well. This is, in part due to competition for the limited amount of LPL, for which big CM are the preferred substrate (3). This competition accounts for the strong correlation between fasting plasma triglyceride levels and the amount of alimentary lipemia brought on by consuming fat. Even if the rate of VLDL secretion (2) may not change, their catabolism does, leading to accumulation (4). Moreover, under normal metabolic conditions, binding of more apoE is made possible by the larger size of chylomicron remnants and the lower surface area of apoB48 vs. apoB100 when compared to VLDLs. These factors lead to increased hepatic reuptake and a shorter plasma residence time (minutes for CM vs hours for VLDL). B: apoB48 kinetics are such that they represent only 1-2% of apoB100 in the circulation. Low steady state concentrations could be caused by large fluxes. This is the case when we contrast serum amounts of apoB100 versus apoB48, as demonstrated in **(B)** apoB48 doubles during the postprandial phase but represents only 1% to 2% of apoB100, primarily due to the fact that VLDL life is measured in hours and LDL’s in several days and most apoB100 is in LDL. As shown in **Figure 5** elegant kinetic studies utilizing stable isotopes in humans indicate a daily flux of 50 plasma pools of chylomicrons. These are far slower than the fluxes of VLDL, which are 10 pools per day for VLDL1, 3.8 pools per day for VLDL2, and 2 pools per day for IDL.

Why does VLDL increase in the postprandial period, when most TG are being transported by CM in this phase as we have just indicated? Indeed, 70-90 g of TG/day (about to 100 mmoles) travel in CM whereas VLDL carries 20-30 g/day (about to 30 mmoles). Increases in apoB48 and apoB100-containing VLDL are what cause the increase in particle number, even though dietary fat in CM accounts for around 80% of the rise in plasma triglyceride levels after a meal (8, 39). One must bear in mind that there are far more TG per apoB molecule (indicator of particle number) in CM than in VLDL. A key concept that has great pathophysiological significance is illustrated in **Figure 6A**. The reason for postprandial increases in blood levels of VLDL may be due to competition for the limited amount of LPL, for which large CM are the preferred substrate. This competition accounts for the strong correlation between fasting plasma triglyceride levels and the amount of alimentary lipemia brought on by consuming fat (8, 18, 33, 102). Particularly, under normal metabolic conditions, binding of more apoE is made possible by the larger size of chylomicron remnants and the lower surface area of apoB48 vs. apoB100 when compared to VLDL. These factors lead to increased hepatic absorption and a shorter plasma residence time (minutes for CM vs hours for VLDL). Normally, the metabolic products of plasma chylomicron metabolism are large, lipid-rich, and buoyant, but as VLDL remnants gradually degrade, IDL particles are produced, which later develop into LDL. In a nutshell: more particles but less TG.

The huge difference in residence times and catabolic rate sometimes obscure the key role of fluxes when analyzing lipoprotein metabolism. Nature follows what Heraclitus favored: everything is in a state of flux. Large fluxes may be behind low steady state concentrations. For instance, as shown in **Figure 6B**, this is the case when we compare serum concentrations of apoB100 vs apoB48. ApoB48 is only 1-2% of that of apoB100, mainly because VLDL has a life of hours and LDL of days. There is a daily flux of 50 plasma pools of chylomicrons, according to a recent elegant kinetic investigation using radioisotopes in humans as shown in **Figure 5**. When compared to the fluxes of VLDL, which are 10 pools per day for VLDL1, 3.8 pools per day for VLDL2, and 2 pools per day for IDL, these are substantially slower (21, 39).

### 6.2 Remnants of chylomicrons contain more cholesterol than LDL and are regarded as more atherogenic

In combination, lipolysis by LPL and other lipases results in remnants, which are particles with primary structural proteins that are maintained (23, 25, 103). As already indicated, these proteins are apoB48 (CM) and apoB100 (VLDL). In the case of VLDL remnants, these are made up of a class of particles known as intermediate-density lipoproteins (IDL), which have been shown to have distribution overlap with small VLDL particles with d 1.006 g/ml and Sf 20-60. IDLs have a density (d) of 1.006-1.019 g/ml and a Sf of 12-20.

All particles in this density range (d= 1.006 g/ml) contribute to the remnant spectrum to varying degrees, even though there are only a few unaltered nascent VLDL and CM present at any given moment due to LPL rapid activity. This complexity highlights the challenges currently encountered in reliably measuring remnant particles, as we will explore later in section 9.

**Figure 7:**
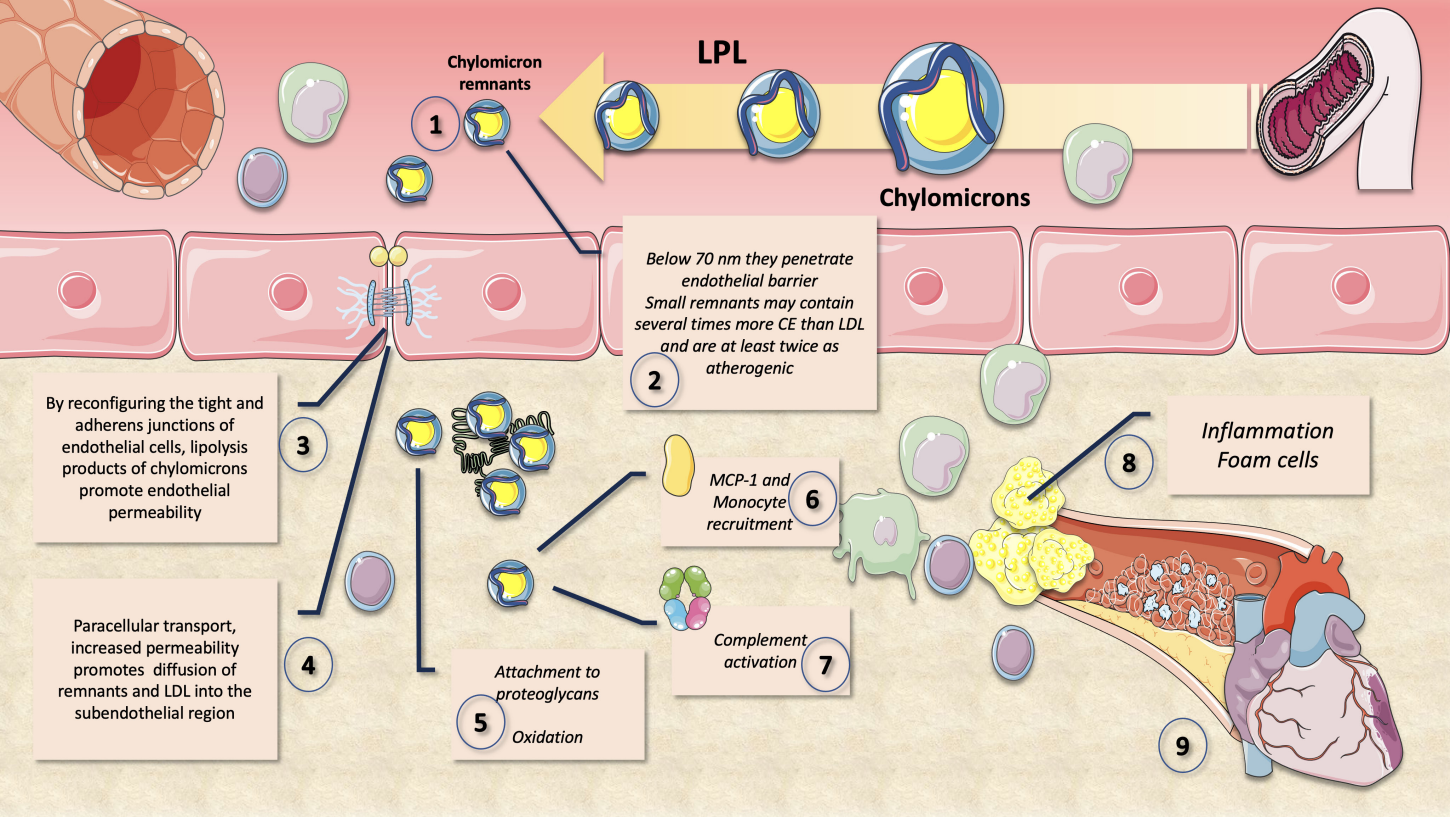
Atherogenic effects of chylomicron (and VLDL) remnants. After extensive lipolysis, chylomicron remnants of less than 70 nm diameter (1) may pass through the endothelium barrier (2). Small remnants are at least twice as atherogenic and can contain many times more CE than LDL. Lipolysis products of CM increase endothelial permeability (3) and their own ingress (4) to the intima by rearranging the tight and adherens junctions of endothelial cells. Their apoE and apoB48 bind proteoglycans facilitating accumulation and oxidation (5). Monocyte recruitment is facilitated by MCP-1 (6), complement activation is enhanced (7) and overall inflammation and foam cell formation (8) leading to atheroma (9).

As we illustrate in **Figure 7**, one important factor raising the risk of ASCVD is the capacity of cholesterol-enriched residual lipoproteins to transfer their lipid cargo to macrophages, which are the cells responsible for cholesterol deposition in arterial plaques (25, 31, 104). It is important to remember that a remnant particle may carry more cholesterol than an LDL particle. Due to their pro-inflammatory effects, which are more severe per particle than those of modified LDL, as well as their unregulated uptake by macrophage scavenger receptors, remnant lipoproteins (RLP) that have undergone oxidative alteration play a substantial role in atherogenesis. Due to their capacity to transfer cholesterol to the artery wall, RLP with a diameter of less than 70 nm are able to contribute to atherosclerosis (73, 105–108). According to numerous reports, CM remnants can pass through the artery wall and are then trapped in the subendothelial area (109, 110). Fully hydrolyzed CM remnants may be preferentially retained in comparison to other lipoproteins and have a cholesterol content that may be several times higher than that of LDL particles (111, 112). The fact that CM remnants contribute to the cholesterol accumulation within the intima is therefore not surprising.

Moreover, by reconfiguring the tight and adherens junctions of endothelial cells, remnants have been found to promote enhanced endothelial permeability (**Figure 7**). Through paracellular transport, increased permeability may in turn help to promote the diffusion of remnants, sd-LDL and LDL into the subendothelial region (106, 107).

A crucial aspect of atherosclerotic progression is inflammation, which necessitates that monocytes attach to endothelial cells and infiltrate the arterial wall. Monocyte chemoattractant protein-1 (MCP-1) is demonstrated to be expressed in mRNA and protein form when CM remnants are present. MCP-1 increases monocyte migration and is therefore essential for the growth of atherosclerosis. It has also been shown that exposure to CM remnants causes endothelial cell apoptosis, as well as an increase in the production of plasmin activator inhibitor-1 (PAI-1), a key regulator of thrombus formation, in endothelial cells (73, 106, 107).

On top of those atherogenic traits, remnant lipoproteins also carry thrombogenic substances, activate monocytes, and promote the death of endothelial cells and the proliferation of smooth muscle cells. Because they have been shown to enhance monocyte adherence to endothelial cells and to produce inflammation by alternative inflammasome activation, their increased levels of apoCIII may also have atherogenic effects. Finally, remnants’ prolonged plasma residence time makes them susceptible to additional changes such as glycation, which can raise their atherogenicity. To stop the evolution of atherosclerotic CVD, it is essential to comprehend how CM metabolism is regulated and how CM remnant accumulation relates to CVD risk in insulin resistance conditions (73).

### 6.3 Cross-regulation of LPL in addition to that of apoCII/CIII causes postprandial TG to be diverted to adipose tissue.

Along with the previously mentioned regulation of LPL activity, there is also another level of sophistication in the control of LPL activity in diverse tissues, that facilitates the body’s ability to physiologically split the TRL load in accordance with its demands. Basically, during a fast, oxidative tissues like the heart and skeletal muscle preferentially take up lipids, while adipocyte storage is not favored. On the other hand, LPL activity increases significantly in adipocytes after eating while decreasing in oxidative tissues. It is now clear that LPL tissue-specific cross-regulation is essential for this trafficking and partitioning of TG.

#### 6.3.1 ANGPTL3, ANGPTL4, and ANGPTL8 are tissue-specific regulators of lipolysis

Following a meal, LPL activity rises in white adipose tissue (WAT) but falls in muscles. In contrast, during a fast, LPL activity reduces in WAT but rises in muscles. Although, as we shall see, tremendous progress has been made in recent years, the mechanism controlling tissue-specific LPL activity throughout the fed-fast cycle much remains to be determined. For LPL activity to respond to constantly changing metabolic circumstances, tissue regulation is necessary. ANGPTL3, ANGPTL4, and ANGPTL8 have been discovered as important tissue-specific regulators of lipolysis (113–116).

In short, ANGPTL3 (secreted continuously from the liver), inhibits lipoprotein lipase in muscle and the heart during the postprandial period, through an endocrine mechanism. In contrast, when fasting, ANGPTL4 released by adipocytes inhibits lipoprotein lipase in adipose tissue in a paracrine manner.

**Figure 8:**
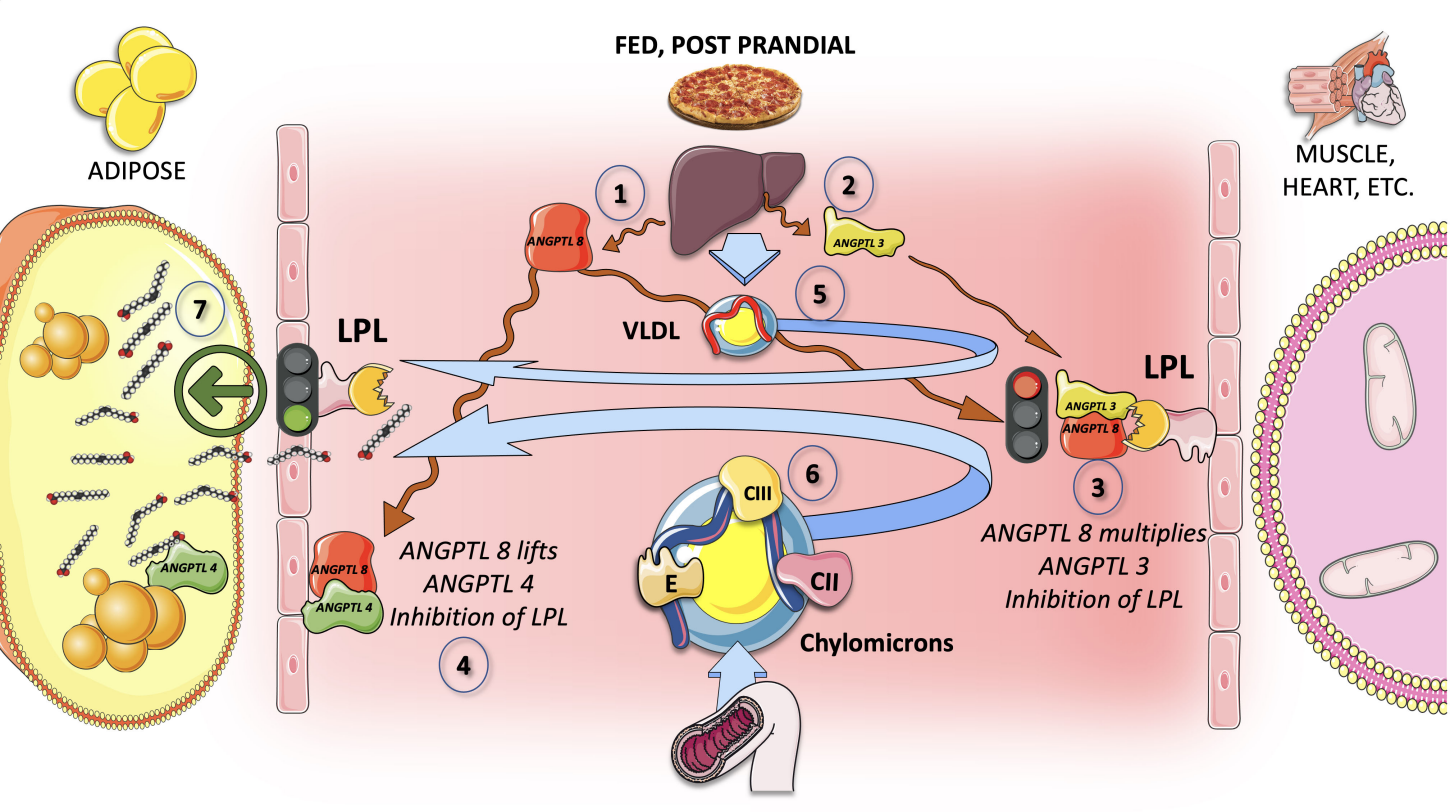
Chylomicron TG fluxes are split in the postprandial period by the axes ANGPTL 3, 4, and 8. In addition to the previously detailed regulation of LPL activity, there is a more precise control of the enzyme in various tissues that enables the physiologic division of the TRL load according to the requirements of the body. Basically, during a fast, oxidative tissues like cardiac and skeletal muscle preferentially uptake lipids, while adipocyte storage is not preferred. Conversely, LPL activity is significantly increased in adipocytes after feeding while being decreased in oxidative tissues. In the postprandial period, the liver secretes ANGPTL8 (1) on top of the ANGPTL3 (2) that has a more constant secretion throughout the day. ANGPTL8 strongly enhances ANGPTL3 inhibition of muscle LPL (3). Simultaneously, ANGPTL8 binds ANGPTL4 to release its inhibition of adipose tissue LPL (4). ANGPTL4 acts in a paracrine fashion to shift TRL away from adipose during fasting, allowing energy to flow to oxidative tissues. The overall result is that both VLDL (5) and CM (6) are now preferentially taken up by adipose tissue. The postprandial liver release of ANGPTL8, which also lifts ANGPTL4’s inhibition on adipose tissue LPL, is essential for the cycle’s fine regulation. As a result, fat is preferentially sent to adipose tissue for storage after a meal. This regulation is a fine-tuning of the regulation of insulin itself as well as the ratio of apoCII/apoCIII in CM and VLDL.

The discovery of ANGPTL4 and ANGPTL3 has greatly improved our understanding of this mechanism because they are both potent LPL inhibitors (117–120). The most recent hypotheses state that ANGPTL8 (secreted postprandially) stimulates ANGPTL3 in an endocrine way to reduce LPL activity in the heart and skeletal muscle, while ANGPT4 suppresses LPL activity in WAT by interacting with intracellular and circulating species (**Figure 8**). Fasting raises ANGPTL4 but lowers ANGPTL8, which lowers LPL activity in WAT and raises it in muscles, respectively. TG are consequently directed toward the muscles for oxidation. Eating, on the other hand, decreases ANGPTL4 activity due to ANGPTL8 attenuation of its inhibitory effect, increasing LPL activity in the WAT while decreasing it in the muscles, directing circulation of CM and VLDL to the WAT for storage (113–116, 120–122).

ANGPTL3 and ANGPTL8 are more readily secreted in response to insulin, and ANGPTL8 levels rise when food is consumed. **Figure 8** illustrates how the presence of ANGPTL8 in adipose increases TRL-triglyceride flow to adipose tissue during the postprandial state (when insulin levels are high) while reducing LPL activity in muscles.

The importance of ANGPTL3 in this process, as well as findings from animal and human loss of function investigations, have shown the potential involvement of ANGPTL3 inhibitors as a treatment option for hypertriglyceridemia, as we discuss later in section 10 of this review (30, 118, 123).

#### 6.3.2 ANGPTL 8 as a master regulator?

The relevance of LPL in the body’s overall energy economy has sparked some speculation about the involvement of these regulators in evolution. Indeed, low calorie consumption presented a severe threat to human existence during the evolution of humans. According to the thrifty gene theory, individuals with certain genes, survived famines by accumulating more fat, and as a result, evolutionary selection favored genes and genetic variants that resulted in the accumulation of adipose storage. ANGPTL8 may be considered a thrifty gene because one of its principal functions is to enhance fat accumulation after a meal (115, 116). Constant feeding increases circulation ANGPTL8 levels, leading to increased adipose storage (fat) and hypertriglyceridemia, and it is not a huge stretch to reckon that the same ANGPTL8 protein that likely safeguarded our ancestors from famine now predisposes humans to metabolic syndrome.

By suppressing ANGPTL8, it may be feasible to reverse thrifty features such as obesity, circulating TG levels, and metabolic syndrome (115, 116, 122). In fact, ANGPTL8 deficiency in mice reduces serum TG levels and obesity. In mice, injection of an ANGPTL8 Ab consistently reduced adiposity and circulating TG levels. Whether this is a workable new strategy to stop this element of metabolic syndrome in humans remains to be seen.

#### 6.3.3 Did nature favor a constant state of inhibition of LPL?

The precise integration of apoCII and apoCIII’s and the ANGPTL axis’s control of LPL is still unclear. As was made obvious in the discussion above, significant advancements have been made in the description of these two paths, but there are still many gaps that, when filled, will enable a more complete comprehension of the process overall.

If we adopt an evolutionary perspective for these processes, for instance, access to fatty foods in the distant past required prolonged energy expenditure (hunting). Consequently, ApoCIII loss-of-function mutations became very uncommon in populations, which may be accounted for by the requirement for a long TRL circulation period when dietary fat is sparse, as it has been for a significant amount of human history (21, 24, 46). When apoCIII is very low, this circulation time is less than 8 minutes for CM whereas it is on the order of 30 minutes under normal circumstances as we depict in **Figure 6**. It appears that, to retain a circulating mass of TG available before the next meal and for as long as feasible, nature favored or preferred a constant state of inhibition of LPL. The important role that apoCIII plays as an LPL inhibitor as well as the findings from studies on animal and human loss of function have also shown the potential use of apoCIII inhibitors as a treatment approach for hypertriglyceridemia, as we summarize in section 10.

### 6.4 Spillover as a postprandial source of FA for VLDL

A portion of the FA issued from CM by LPL is washed into the bloodstream rather than being absorbed into tissues. Spillover is the term for this route and is shown in **Figure 3**. This postprandial input of dietary FA may account for 5–20% of FA to the plasma pool after a meal (21, 116, 124). Given that dietary FA are re-esterified and packed as VLDL-TG, it is possible that dietary FA spillover contributes to the postprandial rise in circulating liver-derived VLDL. Any defect or delay in CM turnover may not only favor VLDL increases (by competition for LPL) but provide substrate for more VLDL production by the liver, by virtue of the high insulin levels in the postprandial period.

## 7 Postprandial lipid metabolism: hormonal control

Although intestinal lipid absorption and lipoprotein secretion were formerly believed to be relatively passive routes, they are now understood to be complicated, controlled processes. The considerable adjustments in gut hormone secretion and remission of T2D after gastric bypass surgery emphasize even more the crucial part the intestine plays in controlling metabolism (69).

### 7.1 Incretins modulate insulin secretion and chylomicron metabolism

Glucagon-like peptide-1 (GLP-1) and glucagon-like peptide-2 (GLP-2), two hormones released in equal levels from enteroendocrine L-cells after food intake, paradoxically have opposing effects on intestinal lipoprotein production, as shown in **Figure 4**. GLP-2 administration has been demonstrated to promote the secretion of both stored TG and preformed CM, whereas GLP-1R agonists have been shown to limit CM output in animal and human investigations as we detail below.

#### 7.1.1 GLP-1

A powerful incretin, GLP-1 mediates several effects that control glycemia, including glucose dependent insulin secretion. To take advantage of these positive benefits, pharmaceutical drugs have been developed that either limit endogenous GLP-1 degradation by inhibiting dipeptidyl peptidase-4 (DPP-4 inhibitors such as sitagliptin, saxagliptin, linagliptin, and alogliptin) or activate GLP-1 receptors (GLP-1R agonists such as dulaglutide, exenatide, liraglutide, lixisenatide and semaglutide). These molecules have also been employed in the scientific community as instruments to clarify the roles and subsequent mechanisms of GLP-1 (125–129). Studies on these compounds in both animals and humans have revealed significant impacts on lipid metabolism in addition to glycemic control. In healthy and T2D subjects, GLP-1-mediated lipid control has also been demonstrated. A GLP-1R agonist dramatically reduced TRL-ApoB48 accumulation in the postprandial state, according to research on normolipidemic, normoglycemic men (130).

There is still much to learn about how GLP-1 controls lipid metabolism. Reduced lipid absorption, decreased CM secretion, and/or improved clearance from the circulation are only a few possible pathways that could explain improvements in postprandial lipemia with GLP-1R agonists and DPP-4 inhibitors. Exenatide (a GLP-1 agonist) or a GLP-1 infusion can also regulate intestinal lipoprotein synthesis without affecting changes in gastric emptying, as was shown by bypassing the stomach (61, 130–133).

By reducing the rate of intestinal lipid absorption and therefore restricting the amount of lipid available within the enterocyte for CM synthesis, GLP-1R activation may control lipid metabolism.

A brain-gut axis has recently been proposed as a theory to explain how GLP-1R agonists affect postprandial lipoprotein synthesis. It has been discovered that GLP-1-producing neurons in the solitary tract of the brain stem send their signals to the arcuate (ARC), paraventricular (PVN), and dorsomedial (DMH) nuclei of the hypothalamus, which express the GLP-1R. According to a recent study, plasma and TRL-TG and TRL-ApoB48 levels in fat-loaded hamsters were decreased when the GLP-1R agonist exendin-4 was injected into the brain’s third ventricle (127, 129).

In addition, the activity of MTTP, which is necessary for the lipidation of apoB48, is decreased by GLP-1R agonists, suggesting another mechanism by which they may function (129, 134, 135).

#### 7.1.2 GLP-2 causes an increase in postprandial lipemia

In humans, GLP-2 causes an increase in postprandial lipemia. According to some evidence, an increase in nitric oxide (NO), a known GLP-2 mediator that increases mesenteric blood flow, may mediate the effects of GLP-2 on postprandial lipids (69).

GLP-1 and GLP-2 thus have opposing effects on the synthesis of intestinal lipoproteins. According to research, when GLP-1 activity is sustained, the effects of GLP-1 take precedence over those of GLP-2 under physiological settings. However, the overall impact of GLP-1 and GLP-2 on intestinal lipid transport is still unknown and should be clarified (69, 130, 131, 133).

## 8 Type 2 diabetes and postprandial metabolism

### 8.1 Type 2 diabetes is associated with increased chylomicron production and catabolism

Despite apparently normal TG levels in the fasting state, postprandial increases of hepatic and intestinal lipoproteins are ostensible in T2D patients (61, 86). A study found that hyperinsulinemic men produced enteral lipoproteins with apoB48 at a higher rate than men with normal insulin levels. T2D patients also showed increased CM synthesis, there was also evidence of decreased CM catabolism (61, 86). Further highlighting the link between insulin resistance and postprandial lipid abnormalities, a recent study found that insulin resistant, abdominally obese adults had higher postprandial TG concentrations than abdominally obese adults without insulin resistance and non**-**abdominally obese controls (61, 86).

Patients with T2D have also been found to have longer duration of postprandial high TG levels. For instance, TG levels peaked at 2 hours after an oral lipid load in healthy controls but remained elevated at 4 hours in T2D patients, further emphasizing the role of CM catabolism in this situation (19, 24, 31, 136).

Dysregulation of insulin signaling at the level of the enterocyte may contribute to postprandial dyslipidemia. In a fructose-fed hamster model of insulin resistance, a study found cellular changes in the insulin receptor signaling pathway as well as a lack of sensitivity to insulin-induced downregulation of CM secretion. Through altered CM assembly, several other changes related to CM oversecretion may take place at the level of the enterocyte (19, 21, 136, 137).

The increased chylomicron synthesis seen in type 2 diabetes is likely caused by a variety of factors.

a) It appears to be significantly influenced by insulin resistance. Hyperperinsulinemic-euglycemic clamps show that type 2 diabetics no longer exhibit the inhibitory impact of insulin on intestine apoB48 lipoprotein synthesis (24, 31, 94).
b) It is also believed that delayed chylomicron degradation contributes to diabetic postprandial hyperlipidemia. Several investigations have reported evidence confirming the presence of impairments in chylomicron catabolism. Reduced fractional catabolic rates of apoB48 have been seen in type 2 diabetics and insulin-resistant obese persons in several kinetic studies (31, 46, 71, 79, 80, 92). The decrease in LPL activity seen in type 2 diabetes may be connected to this. Since insulin activates LPL, a ‘relative’ insulin deficit and/or insulin resistance in type 2 diabetes may be the cause of decreased LPL activity (**Figure 3**).
c) Further, elevated plasma levels of ApoCIII, an inhibitor of LPL, are found in people with type 2 diabetes and non-diabetic insulin resistance, which may also be a factor in the slowed chylomicron catabolism (71, 79, 80, 92).

Individuals with type 2 diabetes have typical abnormalities in the metabolism of intestinally generated lipoprotein even when taking statin medication, according to very recent state-of-the art analysis of the kinetics of lipoproteins containing apoB48. Individuals with type 2 diabetes demonstrated the following characteristics in comparison to control individuals:

a) higher production rates for apoB48 -containing particles secreted in the form of chylomicrons and VLDL.
b) delayed chylomicron lipolysis.
c) accumulation of apoB48 VLDL with a prolonged residence time in the circulation (24, 46).

Chylomicrons, apoB48 VLDL, and their remnants were shown to be twice as high postprandially, due to these alterations in the postprandial response, which may have an impact on atherosclerosis. However, there was a non-significant tendency toward greater mean plasma TG in those with type 2 diabetes, even so, both values were still well within the normal range. It is important to note that the fasting lipid profile did not provide any evidence of a difference between the two groups evaluated here (24, 46). This fact supports the need of dynamic tests for postprandial metabolism as we discuss in Section 9.

These findings have important implications for future research on postprandial lipoprotein structure, metabolism, and particle atherogenicity. New research will help us better understand the causes of residual risk in statin-treated people and, possibly, the outcomes of cardiovascular outcome trials. Higher TG, this enigmatic risk factor, will undoubtedly become more readily apparent and offer fresh opportunities for intervention.

### 8.2 Defect in chylomicron metabolism is already present in IR patients without overt diabetes

Together, the generation of intestinal lipoproteins is a tightly controlled process that is drastically changed by insulin resistance (IR).

In non-diabetic, insulin-resistant, obese individuals, there is a twofold increase in apoB48 synthesis in the postprandial state, according to a lipoprotein kinetic study (39). This investigation found a positive correlation between plasma insulin levels and the synthesis rate of apoB48, indicating that insulin resistance is probably a factor. A large rise in the apoB48 was observed in people with type 2 diabetes in an ***in vivo*** kinetic investigation carried out in the fed state, which was mostly due to a notable increase in the apoB48 production rate (21, 24, 31, 39). Our own studies have evidenced increased fasted apoB48 in obese adolescents (96) and even in lean adolescents with insulin resistance (97), together with elevated sd-LDL (96, 97, 138), apoCIII and ANGPTL3. Isocaloric fructose restriction for only 10 days reduced these markers by 30% (72, 98, 99).

### 8.3 Role of microbiota

The dysbiosis of the gut microbiota in type 2 diabetes may affect intestinal lipid metabolism via a variety of mechanisms albeit human data are still scarce in this area (61).

Increased chylomicron synthesis may result from increased intestinal permeability, fat absorption, and intestinal insulin resistance caused by low-grade inflammation. Reduced GLP-1 secretion, which might result in increased lipid absorption and chylomicron synthesis, may be caused by decreased SCFA production, which has anti-inflammatory properties and stimulates GLP-1 production.

Primary bile acids can be converted by the gut microbiota into secondary bile acids, which boosts the synthesis of GLP-1. The conversion of primary bile acids into secondary bile acids is decreased in dysbiosis of the gut microbiota, which may result in a reduction in GLP-1 synthesis and immediate effects on intestinal lipid metabolism (61, 69, 130).

### 8.4 MTTP and DGAT in diabetes

The lipidation of apoB48 during CM assembly is mediated by MTTP, a binding that stops apoB48 from degrading as a result. In comparison to chow-fed controls, insulin-resistant hamsters displayed higher intestinal *de novo* lipogenesis, increased intracellular apoB48 stability, and increased MTTP mass. As stated earlier, another enzyme involved in TG synthesis in the enterocyte and consequently CM formation is diacylglycerol acyltransferase (DGAT). Additionally, it has been demonstrated that insulin resistance hamster models have increased DGAT activity and expression (40, 42). Through activation of different FA transporters, enhanced dietary lipid absorption may also be enhanced in insulin resistance situations to supply more substrates for CM assembly.

## 9 Clinical and research testing of postprandial lipoprotein metabolism

**Figure 9:**
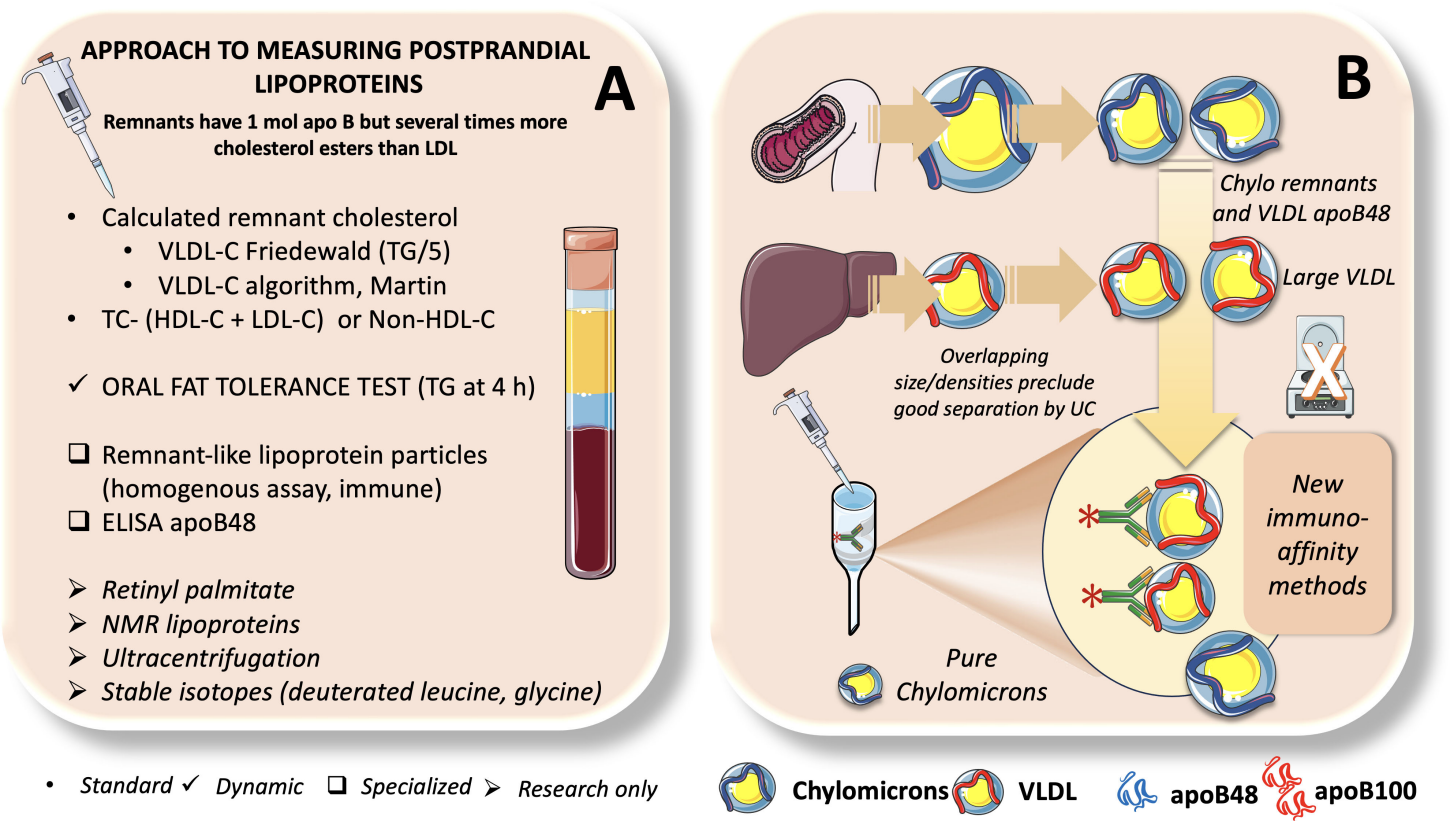
Approaches to measure postprandial lipoproteins. In **(A)** we summarize different methods to assess postprandial lipoproteins from those currently used in routine laboratory testing, dynamic tests like OFTT, specialized tests of less common use such as remnant-like lipoprotein particles and apoB48 ELISA and finally methods used mostly in research such as ultracentrifugation, NMR, retinyl palmitate and stable isotopes. Study of postprandial lipid metabolism is fraught with methodological issues because as shown in **(B)**, chylomicron remnants overlap with large VLDL when separated by UC. New methods developed by us and others now rely on immunoaffinity retention of apoB100 lipoproteins to produce pure chylomicrons.

The fed state predominates throughout the day with the present eating habits in Western nations; the average person only being in the fasted state for a few hours in the wee hours of the morning. However, the fasting lipid profile has been a common way to gauge the risk of CVD (7, 33, 102, 139). To accurately compute low-density lipoprotein cholesterol (LDL-C) using the Friedewald equation and to limit the variability in TG concentration after meal intake, fasting triglycerides are routinely measured. Furthermore, it has been demonstrated that computed LDL-C changes little in response to meal consumption and that measured and calculated LDL-C are closely associated in both fasting and non-fasting stages. However, a paradigm shift toward measuring lipid parameters in the non-fasting or postprandial (i.e., blood sample measurement at specific time points after a standardized meal) states is taking place. This is because non-fasting TG levels are independently related with cardiovascular events. As we summarize in **Figure 9**, approaches to study postprandial lipoprotein metabolism can be simplified as follows: 1. Standard or routine; 2. Dynamic; 3. Specialized; 4. Research only, albeit there is certain overlap among some of them.

### 9.1 Use of standard lipid values to assess presence of remnants in an indirect way

TG, total cholesterol (TC), high-density lipoprotein cholesterol (HDL-C), and LDL-C are the components of the conventional fasting lipid profile used to determine CVD risk. There is, however, little proof that lipid parameter measures taken when fasting are better than those taken while not fasting. Instead, non-fasting measurements have several noteworthy benefits, such as a more accurate representation of the daily average of plasma lipids, ease of blood sampling for patients, laboratories, and clinicians, and increased patient compliance with lipid testing. Indeed, only half of patients with acute coronary syndrome have fasting dyslipidemia, even though this condition is significantly related with an increased risk of CVD. The TG/HDL-C continues to be a valuable tool that correlates with IR, and the presence of remnants and sd-LDL. Non-fasting total cholesterol less LDL cholesterol less HDL cholesterol can be used to compute “remnant cholesterol.” In non-fasting samples, LDL cholesterol can be measured directly, as is common in clinical laboratories, or it can be calculated using the Friedewald equation if the TG level is below 4.5 mmol/L. A classical and simple test to determine their presence of large chylomicrons is to leave plasma overnight in the refrigerator, where the CM float as a creamy supernatant (11).

### 9.2 Dynamic studies: oral fat tolerance testing

In contrast to fasting TG levels, postprandial TG levels (usually at 4 h), which are acquired after having a standardized high-fat meal, are a stronger predictor of coronary artery disease (10, 140). In the context of primary prevention, non-fasting lipid testing has been recommended by several clinical guidelines. A more accurate assessment of a person’s ability to metabolize lipids after eating, showing their metabolic efficiency, can be obtained by analyzing their postprandial lipid profile.

Practical issues, such as the need to prepare an OFTT meal, collect many blood samples, spend several hours in the hospital, and the absence of defined clinical guidelines. Additionally, there are currently no management guidelines for this kind of hypertriglyceridemia that have been accepted internationally. Nevertheless, a few studies have found that non-HDL-C, which is made up of LDL-C, TG-rich remnant lipoproteins, and lipoprotein (a), provides a more accurate indication of the risk of developing atherosclerotic cardiovascular disease (ASCVD) than LDL-C alone, particularly in individuals with hypertriglyceridemia (13, 102, 141, 142)

OFTT, a comparable technique to evaluate lipid parameters at predetermined intervals after consuming a high-fat meal, is not yet commonly used in clinic settings to assess the effectiveness of lipid metabolism.

#### 9.2.1 Standardized test for fat tolerance

After an 8-hour fast, a test meal containing roughly 75 g of fat is consumed, and 4 hours later, a single TG measurement should be taken as part of the standardized fat tolerance test. The 1-point technique may be helpful for clinical testing and investigations with a high number of participants. The OFTT meal should be simple to make and comprise mixtures of digestible saturated and unsaturated fatty acids. Commercially available whipped cream or cream cheese, each of which contains about 250 g of calories and 15 g of sugar (whipping cream contains roughly 10 g of protein and 10 g of carbohydrates) have been recommended (11).

The Expert Panel’s 2011 statement on postprandial hypertriglyceridemia advised measuring TG 4 hours after having an OFTT meal, which included 75g of fat, 25g of carbohydrates, and 10g of protein. This test was advised for persons with fasting TG concentrations between 89 and 175 mg/dl (1-2 mmol/l). An ideal postprandial response was determined as TG concentrations 2.5 mmol/L (220 mg/dL) at all time-points after this OFTT meal. The predicted TG values mentioned in our previous Expert Panel statement were validated by two recent trials (CORDIOPREV and GOLDN), each of which included over 2,000 patients (11).

#### 9.2.2 When should a test for fat tolerance be conducted?

In patients suspected of having additional metabolic co-morbidities, the Expert Panel advises administering a fat tolerance test in those with non-fasting TG levels of 1.3-2.3 mmol/L (115-200 mg/dL) or fasting TG values of 1-2 mmol/L (89-175 mg/dL).

#### 9.2.3 Test for fat tolerance in diabetes mellitus

The best way to assess postprandial hypertriglyceridemia in DM patients appears to be to measure postprandial TG at 4 hours after eating (102, 139). Even in the face of normal fasting TG levels, postprandial hypertriglyceridemia has been associated with higher ASCVD morbidity and mortality in individuals with DM. Individuals with DM and non-fasting TG levels between 1.3 and 2.3 mmol/L (115 and 200 mg/dL) or between 1-2 mmol/L (89 and 175 mg/dL) may benefit from an OFTT, whereas those with fasting TG levels below 1 mmol/L (89 mg/dL) may experience exaggerated postprandial hypertriglyceridemia, especially those with diabetic nephropathy (70).

#### 9.2.4 Evidence provided by OFTT

For the past 30 years, studies on human subjects have looked at postprandial lipid reactions to meals high in fat. The amount of fat taken, alcohol use before or during the meal, the presence of other macronutrients, the presence of fiber, and physical activity are just a few of the variables that influence how the TG reacts to a meal that contains fat (18, 70, 133, 141).

Numerous prospective studies examining this connection have further reinforced the commonly established theory about the impact of non-fasting lipids to CVD risk. Postprandial TG levels, but not fasting TG levels, were independently linked with incident cardiovascular events in women throughout the course of a 11.4 year follow-up study (143). A relationship between non-fasting TG levels and an elevated risk of coronary heart disease, ischemic stroke, myocardial infarction, ischemic heart disease, and death has been found by further prospective investigation (144). A substantial connection between non-fasting TG and total mortality has been shown, as well as a significant association between non-fasting TG and cholesterol and myocardial infarction risk (6, 18, 33, 102).

Several gaps still exist in our understanding of the dysregulation in intestinal lipid metabolism in insulin resistance conditions and its relationship to CVD. To guarantee the best possible comparability between test subjects, assessment of the functional postprandial lipid profile—i.e., lipid measures at specific time points after a typical meal—is the preferred approach. More research is needed to develop standard procedures that can differentiate between healthy and at-risk populations, including population-specific meal sizes, nutrient composition, blood sampling time points, and markers to measure, as OFTT methodology is still largely unstandardized. Furthermore, robust reference values, which are crucial for appropriately interpreting postprandial parameters, have yet to be developed. These reference values must, however, be unique to the OFTT methodology being used. It is crucial to develop more efficient methods to evaluate metabolic abnormalities that raise CVD risk as the obesity and diabetes epidemics sweep the world.

### 9.3 Specialized assays

#### 9.3.1 Remnant-like lipoproteins

Commercially available kits exist to quantify remnant-like particles. One of them employs an immunoaffinity gel with immobilized anti-apoAI monoclonal antibodies and a special anti-apoB-100 monoclonal antibody that does not bind apoE-rich apoB-100 lipoproteins (105, 106, 139, 145, 146).

The serum remnant lipoprotein cholesterol (RemL-C) assay, which is more modern, uses phospholipase-D and surfactant to directly solubilize remnants. In under 10 minutes, an automated analyzer can complete this assay. Examining postprandial RLP-C in populations with different cardiometabolic conditions might give an idea of the magnitude and duration of RLP-C in the circulation after a meal, even if RLP-C measurements detect remnants of both intestinal and hepatic origin (11, 106, 139, 140, 145, 146).

#### 9.3.2 ApoB48 ELISA

A more comprehensive picture of pro-atherogenic remnant accumulation can be obtained by measuring TRL remnants and apoB48, which is a measurement of CM particle number. As stated earlier, apoB48 is a protein that is unique to intestinally produced lipoproteins in humans, it enables a direct determination of CM. Each CM or CM remnant particle contains one apoB48 molecule that does not transfer to other lipoproteins, making apoB48 a reliable indicator of the quantity of CM particles. Sandwich ELISA kits that are sold commercially can be used to measure apoB48 (39, 139, 145). Lately, chemiluminescence enzyme immunoassays have been developed for automated measurement (147, 148).

In the fasting state, apoB48 makes up a relatively minor portion of overall apoB (**Figure 6**). The proportion of apoB48 in relation to apoB100 is still very small, even in the postprandial state when there is an increase in the number of apoB48-containing lipoproteins from the intestine.

Notwithstanding its value, apoB48 testing alone cannot fully describe postprandial lipoprotein alterations. VLDL synthesis also increases in the postprandial phase, which apoB48 testing does not show. Likewise, as previously indicated, due to preferential lipolysis of CM over VLDL, there is a larger tendency for VLDL to accumulate postprandially. Following a moderate fat load, apoB100-containing particles typically account for 80% of the increase in TRL particle number, while apoB48-containing particles account for only 20% of the increase (39).

### 9.4 Research only approaches

#### 9.4.1 Retinyl palmitate

In postprandial lipoprotein (PPL) investigations, retinyl-palmitate (RP) has frequently been employed as a marker for CM. With the meal, a loading dosage of vitamin A is administered. Dietary sources of vitamin A are transformed into retinol in the gut, then re-esterified to retinyl esters, principally RP, in the intestinal mucosa, and then integrated into lymph CM (139). The concentration of RP in CM without a loading dose of vitamin A is typically relatively low. RP stays within CM and their remnants as they travel through lipolysis on their way to the liver. After being absorbed by the liver, CM remnants become RP, which is then hydrolyzed, re-esterified, and stored as esters inside hepatocytes. Although some retinol is secreted bound to retinol binding protein, neither free RP nor lipoproteins produced by the liver contain any secretion into circulation. As a result, RP is a reliable indicator of CM and CM-derived lipoprotein particles, particularly in terms of its capacity to distinguish these lipoproteins from VLDL and its byproducts (139).

The use of this marker for PPL studies comes with three warnings. First, there may be a negligible transfer of RP from CM to other lipoproteins. Uncertainty surrounds the size of this transfer. Second, the pattern of retinyl esters in CM is influenced by the fatty acid composition of the meal, which in turn impacts the amount of RP in CM. Thirdly, consider that RP does not represent entire PPL because it only measures CM/CM remnants and not VLDL/VLDL remnants (24).

#### 9.4.2 Ultracentrifugation

When used to identify common classes of lipoproteins before moving on to the next phase, density gradient ultracentrifugation (DGU) is very effective and is indeed the method that has permitted considerable advances in the field of lipoproteins for over 7 decades.

The limitation is that it is not possible to separate remnants using DGU alone. Undeniably, remnant particles produced by the lipolysis of newly synthesized postprandial lipoproteins, CM and VLDL, cover nearly the whole spectrum of lipoprotein densities (1, 9, 149).

DGU can be employed if the only objective is to isolate parent CM and VLDL and not their remnants, but this does not offer a thorough assessment of PPL. It requires a quick first CM spin that lasts only 30 minutes. To purify the VLDL fraction, the density of the infranatant can then be changed to 1.006 g/mL and a longer spin can be performed. This fraction will exclude VLDL remnants that have undergone lipolysis and re-mobilization of TG from their core, causing them to shrink and become denser. However, the larger CM remnants are contaminants of this fraction.

To quantify remnants, it is necessary to either separate these remnant particles from other like-density lipoproteins by methods other than DGU, or measure a component or a label of remnants that remains with the lipoproteins as they proceed through lipolysis. Serum TG or calculated “remnant cholesterol” can be utilized as broad markers when the goal of the investigation does not require lipoprotein-specific measurements.

#### 9.4.3 Immunoaffinity

Due to limitations in the laboratory techniques employed to separate these particles, research into the relative roles and abundances of CM and VLDL particles in the postprandial state has been hampered. TRL can be separated by ultracentrifugation, and CM and VLDL can be partially separated by it as well. However, ultracentrifugation is unable to efficiently separate particles with overlapping densities, such as small CM or CM remnants from large VLDL particles and VLDL remnants. Because of this technical stumbling block, it has been difficult to characterize the following: 1) the relative contribution of TG from the liver and the intestines to postprandial lipid metabolism; 2) the kinetic aspects, specifically the relative turnover rates of CM versus VLDL particles; and 3) the differential clearance rates of TG; 4) the effect of dietary components on postprandial lipid metabolism and cardiovascular risk, including the role of carbohydrates, fructose, and branched-chain amino acids, among others.

We developed an immunoaffinity technique to separate CM from VLDL (57) to get beyond the aforementioned drawbacks of ultracentrifugation (**Figure 9B**). Other alternative immunoaffinity approaches are available, but because CM are extremely labile, we sought to develop a robust method that would enable most intact CM particles to be retrieved (31, 57, 62). As has long been predicted in the literature, intact particles bare information on the tissue of origin and may enable the study of human intestine DNL. We created polyclonal antibodies against an ApoB100 C-terminal region that is missing the apoB48 sequence. This made it possible to separate CM and VLDL particles with great purity (57).

With the aid of this technique, studies to compare how much the small intestine and liver contribute to postprandial lipid fluxes are now available, allowing to answer crucial questions about the impact of postprandial lipids on cardiovascular risk, for example.

With the aid of our method, we produced preliminary evidence indicating that enteral conversion of dietary sugar to fat via DNL can be assessed using this technology by employing stable isotope tracer feeding (57, 72).

#### 9.4.4 Stable isotopes

Stable isotope techniques can be used to determine the kinetics of postprandial lipoproteins, including both their lipid and apoprotein components. For three decades, this secure methodology has been in use. It is predicated on the idea that stable isotopes of an element of interest have similar chemical characteristics but distinct atomic masses, allowing mass spectrometry to detect them (24, 31, 39, 55, 56, 150, 151). The assumption is that the compound being tracked by the stable isotope tracer experiences the same metabolic fate. There are numerous tracer types and mathematical models employed. The kinetics of apoproteins like apoB48, apoB100, and apoprotein AI (apoAI) in the fasting and fed states are determined using stable isotopes of amino acids such leucine and glycine. To analyze various FA and TG metabolic fates, stable isotopes of albumin-bound fatty acids, such as palmitic and oleic acids, and of glycerol, are utilized. Special innovations have been created to research the postprandial phase, which is a metabolic non-steady state when several short-lived dynamic alterations take place. The use of mass isotopomer distribution analysis (MIDA) has considerable enhanced the performance of the technique (55, 56, 150). For instance, it was conceivable to compare the extraction of TG comparing adipose and muscle by giving various stable isotopes orally and by intravenous injection to label CM-TG and VLDL-TG, respectively. In contrast to static assessments of lipoproteins, stable isotope technology gives dynamic information on generation (both source and amount) and fractional catabolic rates of lipoproteins (24, 31, 56, 136). The adoption of this methodology is, however, constrained by the high cost of tracers and mass spectrometry. Nevertheless, it remains the gold standard to dissect the complexity of lipoprotein fluxes.

#### 9.4.5 Overall assessment

In conclusion, every method has benefits and drawbacks and yields data that vary significantly. The results from different studies can vary because no approach has been standardized (apart from TG and calculated “remnant cholesterol”). When attempts are made to compare one study to another, this instigates problems. Continued work is required to (a) standardize postprandial lipoprotein (PPL) testing procedures and metrics, and (b) evaluate PPL more comprehensively rather than focusing on discrete factors. Indeed, research on PPL has not yet utilized the full power of proteomics, lipidomics, genomics, and metabolomics within the context of systems biology, albeit great advances are being produced currently.

## 10 New approaches to treat hypertriglyceridemia

In keeping with what has been said in this review thus far, we should first clarify what we mean when we talk about triglyceride lowering therapy. Instead of simply decreasing triglycerides, the therapy should try to minimize the amount of circulating TRL remnants.

In contrast to the previous generation of triglyceride-lowering drugs (including fibrates, nicotinic acid, and omega-3 PUFAs), the novel therapeutics that use antibody and RNA-silencing technologies are highly specific and exhibit improved triglyceride-lowering properties (27, 152, 153).

Triglyceride levels can be reduced by up to 40% in patients with severe hypertriglyceridemia when taking niacin, fibrates, and omega-3 fatty acids.

### 10.1 Fibrates

Fenofibrate did not significantly improve ASCVD outcomes in the type 2 diabetes individuals who participated in the FIELD (154–156) and ACCORD (157–159) trials, but subgroup analysis revealed that these patients had a much lower risk of developing the condition. According to the recently concluded major PROMINENT study, pemafibrate, a selective PPAR agonist, is not protective against ASCVD in patients with increased triglyceride levels and type 2 diabetes (157–159). This finding should put an end to a protracted discussion concerning the clinical benefits of this therapy strategy (51, 94, 103, 152).

### 10.2 EPA and DHA

Omega-3 PUFA preparations have been tested in large ASCVD outcome studies that, overall, have only provided weak evidence of their benefits in reducing ASCVD risk. The REDUCE­IT trial of high-dose eicosapentaenoic acid (EPA), however, reported a 28% relative risk reduction (160–162). This benefit was not replicated in the STRENGTH research (161). These contradictory findings have sparked an ongoing discussion concerning the underlying mechanism underlying the advantages of EPA seen in REDUCEIT. Numerous studies that have been published in the last five years suggest that the mineral oil used as the REDUCEIT trial’s placebo comparator may have slightly influenced how much of a risk reduction the EPA group perceived relative to the control group (27, 153, 161).

### 10.3 Statins, PCSK9 inhibitors, and ezetimibe

These agents that are so effective in the reduction of LDL-C, are however regarded as only moderately effective for the reduction of TG (usually a 5–15% reduction) (26, 141).

### 10.4 ApoCIII and ANGPTL3 are hot therapeutic targets

Over the past five years, research into creating compounds to treat hypertriglyceridemia has focused in increasing TRL lipolysis. Due to their recognized functions in controlling LPL activity and the availability of genetic data that supports the proof of concept that decreasing the concentration of these targets is probably going to result in decreased ASCVD risk, ApoC­III and ANGPTL3 (discussed later) are currently the primary targets. The two most promising methods are those that use RNA silencing and monoclonal antibody inhibition (80, 92). In phase II and phase III clinical trials, antisense oligonucleotides targeting APOC3 mRNA (volanesorsen and olezarsen) are now being investigated (163–165).

#### 10.4.1 The special case of Type 1 chylomicronemia or familial chylomicronemia syndrome

FCS is a rare recessive genetic condition that is frequently misdiagnosed and may have serious clinical repercussions as its primary consequence is acute pancreatitis (166). New therapeutic approaches may ensue from the advancements in our understanding of the roles of apo CIII and angiopoietin- ANGPTL3 (167, 168). Homozygous or compound heterozygous mutations in LPL, apolipoprotein CII, apolipoprotein AV, GPIHBP1, and LMF are the causes of FCS. It has been demonstrated that antisense oligonucleotide targeting apoCIII significantly lowers triglyceride levels even in FCS (169), and this medication is the first one that is currently available for these patients (163–165). Although ANGPTL3 inhibitors have not yet been studied in FCS patients, they have a notable hypotriglyceridemic effect in the milder and more common polygenic variants (32, 164, 170).

In both animal and human investigations, these substances (apoCIII and ANGPTL3 inhibitors) lower plasma levels of apoC­III and ANGPTL3 by about 70% and 80%, respectively (167–169). Evinacumab, a monoclonal antibody that targets ANGPTL3, has been demonstrated to lower plasma triglyceride and LDL-C levels in people with homozygous familial hypercholesterolemia (30, 118, 123). This studies imply that evinacumab may function differently from other drugs that stimulate LDL receptor activation. Microsomal triglyceride transfer protein (MTTP) inhibitors such as mipomersen, if proven safe might also be used to treat these two additional targets (164, 170, 171).

## 11 Conclusions and perspectives

Improving the clinical evaluation of postprandial dyslipidemia is imperative, as it is becoming more widely acknowledged as a significant factor in the onset of atherosclerosis and subsequent cardiovascular disease. Further investigation is important to comprehend the mechanisms that underlie postprandial dyslipidemia, chylomicron metabolism, and their connections to insulin resistance. A more complete understanding of the underlying pathobiology will enable the development of standardized methodologies and biomarker profiles for application in clinical practice for the timely and accurate identification of individuals at risk for CVD. Some of the numerous issues that require in-depth investigation in the upcoming years are: a) the nature hormonal and nutritional factors that influence ANGPTL3, 4, 8 activities and what long-term consequences may be anticipated if their regulation is pharmacologically disrupted; b) the fine tuning of the integration of the regulatory actions of apoCIII, apoAV, and ANGPTL on LPL activity; c) approaches to safe and appropriate treatment of postprandial lipemia; d) uncovering details of the control of remnant production and re-uptake; e) role of microbiota; f) the role of incretins in chylomicron metabolism and the brain-gut axis g) developing of new, standardized and practical methods for remnant assays possibly based on proteomic or lipidomic signatures.

## Author contributions

AG: Conceptualization, Resources, Validation, Visualization, Writing – original draft, Writing – review & editing.

## Glossary

**Table:**

2-MG	2-monoglycerides
ABCG5	ATP-binding cassette G5
ACAT	acyl-coA:cholesterol acyltransferase
ANGPTL	angiopoietin-like proteins
ApoB48	apolipoprotein B48
apoCIII	apolipoprotein CIII
ARC	arcuate nucleus
ASCVD	atherosclerotic cardiovascular disease
ASO	antisense oligonucleotides
CETP	cholesteryl ester transfer proteins
ChREBP	carbohydrate response element binding protein
CM	chylomicron
CVD	cardiovascular disease
DGAT	diacylglycerol acyltransferase
DGU	density gradient ultracentrifugation
DHA	docosahexapentaenoic acid
DM	diabetes mellitus
DMH	dorsomedial nucleus
DNL	*de novo* lipogenesis
EL	endothelial lipase
EPA	eicosapentaenoic acid
ER	endoplasmic reticulum
FA	free fatty acids
FABP	fatty acid binding protein
FABPs	fatty acid-binding proteins
FASN	fatty acid synthase
FoxO1	forkhead box protein O1
G3P	glycerol-3-P
GLP-1	glucagon-like peptide-1
GLP-1R	GLP-1 receptors
GLP-2	glucagon-like peptide-2
GPIHBP1	glycosylphosphatidylinositol­anchored HDL­binding protein 1
HL	hepatic triglyceride lipase
HNF4	Hepatocyte Nuclear Factor 4
HSPG	heparan sulfate proteoglycans
IDL	intermediate-density lipoprotein
LDL	low density lipoprotein
LDLR	LDL receptor
LMF1	lipase maturation factor 1
LOF	loss-of-functions
LPL	lipoprotein lipase
LRP-1	LDL-like receptor protein-1
MC4R	melanocortin-4 receptor
MetS	metabolic syndrome
MGATs	2-MG acyltransferases
MIDA	mass isotopomer distribution analysis
MTTP	microsomal triglyceride transfer protein
NO	nitric oxide
NPC1L1	Niemann-Pick C1-like 1
OFTT	oral fat tolerance testing
PAI-1	plasmin activator inhibitor-1
PPL	postprandial lipoprotein
PUFA	polyunsaturated FA
PVN	paraventricular nucleus
RemL-C	remnant lipoprotein cholesterol
RLPs	remnant lipoproteins
RP	retinyl-palmitate
SREBP-1c	sterol regulatory element binding transcription factor 1c
TG	triglycerides
TRL	triglyceride rich lipoproteins
VLDL	very-low density lipoproteins
WAT	white adipose tissue.

## References

[1] Zilversmit DB. Atherogenic nature of triglycerides, postprandial lipidemia, and triglyceride-rich remnant, lipoproteins. Clin Chem (1995) 41(1):153–8. doi: 10.1093/clinchem/41.1.153 7813071

[2] Zilversmit DB. Atherogenesis: A postprandial phenomenon. Circulation (1979) 60(3):473–85. doi: 10.1161/01.CIR.60.3.473 222498

[3] Nordestgaard BG Zilversmit DB. Large lipoproteins are excluded from the arterial wall in diabetic cholesterol-fed rabbits. J Lipid Res (1988) 29(11):1491–500. doi: 10.1016/S0022-2275(20)38428-5 3241125

[4] Van Heek M Zilversmit DB. Mechanisms of hypertriglyceridemia in the coconut oil/cholesterol-fed rabbit: Increased secretion and decreased catabolism of very low density lipoprotein. Arterioscler Thrombosis (1991) 11(4):918–27. doi: 10.1161/01.ATV.11.4.918 2065043

[5] Pirillo A Norata GD Catapano AL. Postprandial lipemia as a cardiometabolic risk factor. Curr Med Res Opin (2014) 30(8):1489–503. doi: 10.1185/03007995.2014.909394 24673475

[6] de Vries MA Klop B Eskes SA van der Loos TLJM Klessens-Godfroy FJM Wiebolt J. The postprandial situation as a pro-inflammatory condition. Clinica e Investigacion en Arteriosclerosis (2014) 26(4):184–92. doi: 10.1016/j.arteri.2014.02.007 24866730

[7] Nakajima K Tanaka A. Postprandial remnant lipoproteins as targets for the prevention of atherosclerosis. Curr Opin Endocrinol Diabetes Obes (2018) 25(2):108–17. doi: 10.1097/MED.0000000000000393 29493553

[8] Gugliucci A. Triglyceride-rich lipoprotein metabolism: key regulators of their flux. J Clin Med (2023) 12(13):4399. doi: 10.3390/jcm12134399 37445434 PMC10342861

[9] Nakajima K Tanaka A. Atherogenic postprandial remnant lipoproteins; VLDL remnants as a causal factor in atherosclerosis. Clinica Chimica Acta (2018) 478:200–15. doi: 10.1016/j.cca.2017.12.039 29307667

[10] Rathnayake KM Weech M Jackson KG Lovegrove JA. Impact of meal fatty acid composition on postprandial lipaemia, vascular function and blood pressure in postmenopausal women. Nutr Res Rev (2018) 31(2):193–203. doi: 10.1017/S0954422418000033 29547370

[11] Kolovou GD Watts GF Mikhailidis DP Pérez-Martínez P Mora S Bilianou H. Postprandial hypertriglyceridaemia revisited in the era of non-fasting lipid profiles: executive summary of a 2019 expert panel statement. Curr Vasc Pharmacol (2019) 17(5):538–40. doi: 10.2174/1570161117999190517115432 31418346

[12] Borén J John Chapman M Krauss RM Packard CJ Bentzon JF Binder CJ. Low-density lipoproteins cause atherosclerotic cardiovascular disease: Pathophysiological, genetic, and therapeutic insights: A consensus statement from the European Atherosclerosis Society Consensus Panel. Eur Heart J (2020) 41(24):2313–30. doi: 10.1093/eurheartj/ehz962 PMC7308544 32052833

[13] Desmarchelier C Borel P Lairon D Maraninchi M Valéro R. Effect of nutrient and micronutrient intake on chylomicron production and postprandial lipemia. Nutrients (2019) 11(6):1299. doi: 10.3390/nu11061299 31181761 PMC6627366

[14] Mangat R Su J Scott PG Russell JC Vine DF Proctor SD. Chylomicron and apoB48 metabolism in the JCR:LA corpulent rat, a model for the metabolic syndrome. Biochem Soc Trans (2007) 35(3):477–81. doi: 10.1042/BST0350477 17511632

[15] Gugliucci A. Beyond LDL: understanding triglyceride-rich lipoproteins to tackle residual risk. J Clin Med (2023) 12(12):3991. doi: 10.3390/jcm12123991 37373684 PMC10299494

[16] Vallejo-Vaz AJ Corral P Schreier L Ray KK. Triglycerides and residual risk. Curr Opin Endocrinol Diabetes Obes (2020) 27(2):95–103. doi: 10.1097/MED.0000000000000530 32073428

[17] Rikhi R Shapiro MD. Newer and emerging LDL-C lowering agents and implications for ASCVD residual risk. J Clin Med (2022) 11(15):23072319. doi: 10.3390/jcm11154611 PMC9369522 35956226

[18] Nakamura K Miyoshi T Yunoki K Ito H. Postprandial hyperlipidemia as a potential residual risk factor. J Cardiol (2016) 67(4):335–9. doi: 10.1016/j.jjcc.2015.12.001 26744235

[19] Taskinen MR Matikainen N Björnson E Söderlund S Inkeri J Hakkarainen A. Contribution of intestinal triglyceride-rich lipoproteins to residual atherosclerotic cardiovascular disease risk in individuals with type 2 diabetes on statin therapy. Diabetologia (2023) 66(12):2307–19. doi: 10.1007/s00125-023-06008-0 PMC10627993 37775612

[20] González-Juanatey JR Almendro-Delia M Cosín-Sales J Bellmunt-Montoya S Gómez-Doblas JJ Riambau V. Residual risk reduction opportunities in patients with chronic coronary syndrome. Role of dual pathway inhibition. Expert Rev Clin Pharmacol (2020) 13(7):695–706. doi: 10.1080/17512433.2020.1772056 32434452

[21] Borén J Taskinen MR. Metabolism of triglyceride-rich lipoproteins. Handb Exp Pharmacol (2022) 270:133–56. doi: 10.1007/164_2021_520 34676434

[22] Wierzbicki AS Clarke RE Viljoen A Mikhailidis DP. Triglycerides: A case for treatment? Curr Opin Cardiol (2012) 27(4):398–404. doi: 10.1097/HCO.0b013e328353adc1 22565137

[23] Baratta F Cocomello N Coronati M Ferro D Pastori D Angelico F. Cholesterol remnants, triglyceride-rich lipoproteins and cardiovascular risk. Int J Mol Sci (2023) 24(5):4268. doi: 10.3390/ijms24054268 36901696 PMC10002331

[24] Borén J Taskinen MR Björnson E Packard CJ. Metabolism of triglyceride-rich lipoproteins in health and dyslipidaemia. Nat Rev Cardiol (2022) 19(9):577–92. doi: 10.1038/s41569-022-00676-y 35318466

[25] Chait A Ginsberg HN Vaisar T Heinecke JW Goldberg IJ Bornfeldt KE. Remnants of the triglyceride-rich lipoproteins, diabetes, and cardiovascular disease. Diabetes (2020) 69(4):508–16. doi: 10.2337/dbi19-0007 PMC7085249 32198194

[26] Wang K Wang R Yang J Liu X Shen H Sun Y. Remnant cholesterol and atherosclerotic cardiovascular disease: Metabolism, mechanism, evidence, and treatment. Front Cardiovasc Med (2022) 9:913869. doi: 10.3389/fcvm.2022.913869 36324753 PMC9621322

[27] Gouni-Berthold I Schwarz J. New therapeutic approaches for the treatment of hypertriglyceridemia. Herz (2022) 47(3):220–7. doi: 10.1007/s00059-022-05113-x 35451595

[28] Moon JH Kim K Choi SH. Lipoprotein lipase: is it a magic target for the treatment of hypertriglyceridemia. Endocrinol Metab (2022) 37(4):575–86. doi: 10.3803/EnM.2022.402 PMC9449100 36065644

[29] Kadomatsu T Tabata M Oike Y. Angiopoietin-like proteins: Emerging targets for treatment of obesity and related metabolic diseases. FEBS J (2011) 278(4):559–64. doi: 10.1111/j.1742-4658.2010.07979.x 21182596

[30] Kim JY Kim NH. New therapeutic approaches to the treatment of dyslipidemia 1: apoC-III and ANGPTL3. J Lipid Atheroscler (2023) 12(1):23–36. doi: 10.12997/jla.2023.12.1.23 36761060 PMC9884553

[31] Adiels M Matikainen N Westerbacka J Söderlund S Larsson T Olofsson SO. Postprandial accumulation of chylomicrons and chylomicron remnants is determined by the clearance capacity. Atherosclerosis (2012) 222(1):222–8. doi: 10.1016/j.atherosclerosis.2012.02.001 22365426

[32] Gallo A Béliard S D’Erasmo L Bruckert E. Familial chylomicronemia syndrome (FCS): recent data on diagnosis and treatment. Curr Atheroscler Rep (2020) 22(11):63. doi: 10.1007/s11883-020-00885-1 32852651

[33] Nakajima K Nakano T Tokita Y Nagamine T Inazu A Kobayashi J. Postprandial lipoprotein metabolism: VLDL vs chylomicrons. Clinica Chimica Acta (2011) 412(15–16):1306–18. doi: 10.1016/j.cca.2011.04.018 PMC3265327 21531214

[34] Lairon D. Macronutrient intake and modulation on chylomicron production and clearance. Atheroscler Suppl (2008) 9(2):45–8. doi: 10.1016/j.atherosclerosissup.2008.05.006 18595783

[35] Cohn JS. Are we ready for a prospective study to investigate the role of chylomicrons in cardiovascular disease? Atheroscler Suppl (2008) 9(2):15–8. doi: 10.1016/j.atherosclerosissup.2008.05.003 18585099

[36] Black DD. Development and Physiological Regulation of Intestinal Lipid Absorption. I. Development of intestinal lipid absorption: Cellular events in chylomicron assembly and secretion. Am J Physiol Gastrointest Liver Physiol (2007) 293(3):G159–G624. doi: 10.1152/ajpgi.00189.2007 17495031

[37] Cook JR Kohan AB Haeusler RA. An updated perspective on the dual-track model of enterocyte fat metabolism. J Lipid Res (2022) 63(11):100278. doi: 10.1016/j.jlr.2022.100278 36100090 PMC9593242

[38] Steenson S Umpleby AM Lovegrove JA Jackson KG Fielding BA. Role of the enterocyte in fructose-induced hypertriglyceridaemia. Nutrients (2017) 9(4):349. doi: 10.3390/nu9040349 28368310 PMC5409688

[39] Taskinen MR Björnson E Matikainen N Söderlund S Rämö J Ainola MM. Postprandial metabolism of apolipoproteins B48, B100, C-III, and E in humans with APOC3 loss-of-function mutations. JCI Insight (2022) 7(19):e160607. doi: 10.1172/jci.insight.160607 36040803 PMC9675484

[40] Stone SJ. Mechanisms of intestinal triacylglycerol synthesis. Biochim Biophys Acta Mol Cell Biol Lipids (2022) 1867(6):159151. doi: 10.1016/j.bbalip.2022.159151 35296424

[41] Wang Y Zeng F Zhao Z He L He X Pang H. Transmembrane protein 68 functions as an MGAT and DGAT enzyme for triacylglycerol biosynthesis. Int J Mol Sci (2023) 24(3):2012. doi: 10.3390/ijms24032012 36768334 PMC9916437

[42] Buhman KK Smith SJ Stone SJ Repa JJ Wong JS Knapp FF. DGAT1 is not essential for intestinal triacylglycerol absorption or chylomicron synthesis. J Biol Chem (2002) 277(28):25474–9. doi: 10.1074/jbc.M202013200 11959864

[43] McFie PJ Patel A Stone SJ. The monoacylglycerol acyltransferase pathway contributes to triacylglycerol synthesis in HepG2 cells. Sci Rep (2022) 12(1):4943. doi: 10.1038/s41598-022-08946-y 35322811 PMC8943211

[44] Magné J Aminoff A Sundelin JP Mannila MN Gustafsson P Hultenby K. The minor allele of the missense polymorphism Ser251Pro in perilipin 2 (PLIN2) disrupts an α-helix, affects lipolysis, and is associated with reduced plasma triglyceride concentration in humans. FASEB J (2013) 27(8):3090–9. doi: 10.1096/fj.13-228759 23603836

[45] Mahmood Hussain M. A proposed model for the assembly of chylomicrons. Atherosclerosis (2000) 148(1):1–15. doi: 10.1016/s0021-9150(99)00397-4 10580165

[46] Borén J Packard CJ Taskinen MR. The roles of apoC-III on the metabolism of triglyceride-rich lipoproteins in humans. Front Endocrinol (Lausanne) (2020) 11:474. doi: 10.3389/fendo.2020.00474 32849270 PMC7399058

[47] Siri-Tarino PW Krauss RM. Diet, lipids, and cardiovascular disease. Curr Opin Lipidol (2016) 27(4):323–8. doi: 10.1097/MOL.0000000000000310 27389628

[48] D’Aquila T Hung YH Carreiro A Buhman KK. Recent discoveries on absorption of dietary fat: Presence, synthesis, and metabolism of cytoplasmic lipid droplets within enterocytes. Biochim Biophys Acta Mol Cell Biol Lipids (2016) 1861(8):730–47. doi: 10.3380/fendo.2020.00474 PMC5503208 27108063

[49] Closs CI Ruiz Diaz MA Cafferata AM Becú-Villalobos D Nogueira JP. Role of the enterocyte in type 2 diabetes mellitus associated dyslipidemia. Medicina (B Aires) (2018) 78(2):91–8. 29659358

[50] Dash S Xiao C Morgantini C Lewis GF. New insights into the regulation of chylomicron production. Annu Rev Nutr (2015) 35(1):265–94. doi: 10.1146/annurev-nutr-071714-034338 25974693

[51] Santos-Baez LS Ginsberg HN. Hypertriglyceridemia-causes, significance, and approaches to therapy. Front Endocrinol (Lausanne) (2020) 11:616. doi: 10.3389/fendo.2020.00616 32982991 PMC7492386

[52] Laufs U Parhofer KG Ginsberg HN Hegele RA. Clinical review on triglycerides. Eur Heart J (2020) 41(1):99–109. doi: 10.1093/eurheartj/ehz785 31764986 PMC6938588

[53] Nakajima K Tokita Y Tanaka A. Hypothesis II: The majority of VLDL-apoB48 remnants in postprandial plasma are derived from the liver, not from the intestine. Clin Chim Acta (2019) 490:12–6. doi: 10.1016/j.cca.2018.12.010 30553860

[54] Björnson E Packard CJ Adiels M Andersson L Matikainen N Söderlund S. Apolipoprotein B48 metabolism in chylomicrons and very low-density lipoproteins and its role in triglyceride transport in normo- and hypertriglyceridemic human subjects. J Intern Med (2020) 288(4):422–38. doi: 10.1111/joim.13017 31846520

[55] Hellerstein MK Schwarz JM Neese RA. Regulation of hepatic *de novo* lipogenesis in humans. Annu Rev Nutr (1996) 16:523–57. doi: 10.1146/annurev.nu.16.070196.002515 8839937

[56] Hellerstein MK Neese RA. Mass isotopomer distribution analysis at eight years: Theoretical, analytic, and experimental considerations. Am J Physiol Endocrinol Metab (1999) 276(6 39-6):E1146–E1170. doi: 10.1152/ajpendo.1999.276.6.E1146 10362629

[57] Jones GM Caccavello R Palii SP Pullinger CR Kane JP Mulligan K. Separation of postprandial lipoproteins: Improved purification of chylomicrons using an ApoB100 immunoaffinity method. J Lipid Res (2020) 61(3):455–63. doi: 10.1194/jlr.D119000121 PMC7053834 31888979

[58] Schwarz JM Noworolski SM Erkin-Cakmak A Korn NJ Wen MJ Tai VW. Effects of dietary fructose restriction on liver fat, *de novo* lipogenesis, and insulin kinetics in children with obesity. Gastroenterology (2017) 153(3):743–52. doi: 10.1053/j.gastro.2017.05.043 PMC5813289 28579536

[59] Gugliucci A. Sugar and dyslipidemia: A double-hit, perfect storm. J Clin Med (2023) 12(17):5660. doi: 10.3390/jcm12175660 37685728 PMC10488931

[60] Theytaz F de Giorgi S Hodson L Stefanoni N Rey V Schneiter P. Metabolic fate of fructose ingested with and without glucose in a mixed meal. Nutrients (2014) 6(7):2632–49. doi: 10.3390/nu6072632 PMC4113761 25029210

[61] Vergès B. Intestinal lipid absorption and transport in type 2 diabetes. Diabetologia (2022) 65(10):1587–600. doi: 10.1007/s00125-022-05765-8 35908083

[62] Julve J Martín-Campos JM Escolà-Gil JC Blanco-Vaca F. Chylomicrons: Advances in biology, pathology, laboratory testing, and therapeutics. Clinica Chimica Acta (2016) 455:134–48. doi: 10.1016/j.cca.2016.02.004 26868089

[63] Fändriks L. Roles of the gut in the metabolic syndrome: an overview. J Intern Med (2017) 281(4):319–36. doi: 10.1111/joim.12584 27991713

[64] Tomkin GH Owens D. Dyslipidaemia of diabetes and the intestine. World J Diabetes (2015) 6(7):970–7. doi: 10.4239/wjd.v6.i7.970 PMC4499530 26185604

[65] Hayashi AA Webb J Choi J Baker C Lino M Trigatti B. Intestinal SR-BI is upregulated in insulin-resistant states and is associated with overproduction of intestinal apoB48-containing lipoproteins. Am J Physiol Gastrointest Liver Physiol (2011) 301(2):G326–G337. doi: 10.1152/ajpgi.00425.2010 21546579

[66] Hsieh J Hayashi AA Webb J Adeli K. Postprandial dyslipidemia in insulin resistance: Mechanisms and role of intestinal insulin sensitivity. Atheroscler Suppl (2008) 9(2):7–13. doi: 10.1016/j.atherosclerosissup.2008.05.011 18653387

[67] Duez H Pavlic M Lewis GF. Mechanism of intestinal lipoprotein overproduction in insulin resistant humans. Atheroscler Suppl (2008) 9(2):33–8. doi: 10.1016/j.atherosclerosissup.2008.05.013 18676184

[68] Haidari M Leung N Mahbub F Uffelman KD Kohen-Avramoglu R Lewis GF. Fasting and postprandial overproduction of intestinally derived lipoproteins in an animal model of insulin resistance: Evidence that chronic fructose feeding in the hamster is accompanied by enhanced intestinal *de novo* lipogenesis and ApoB48-containing lipoprotein overproduction. J Biol Chem (2002) 277(35):31646–55. doi: 10.1074/jbc.M200544200 12070142

[69] Hein GJ Baker C Hsieh J Farr S Adeli K. GLP-1 and GLP-2 as Yin and Yang of intestinal lipoprotein production: Evidence for predominance of GLP-2-stimulated postprandial lipemia in normal and insulin-resistant states. Diabetes (2013) 62(2):373–81. doi: 10.2337/db12-0202 PMC3554391 23028139

[70] Higgins V Adeli K. Postprandial dyslipidemia in insulin resistant states in adolescent populations. J BioMed Res (2020) 34(5):328–42. doi: 10.7555/JBR.34.20190094 PMC7540238 32934193

[71] Hieronimus B Stanhope KL. Dietary fructose and dyslipidemia: New mechanisms involving apolipoprotein CIII. Curr Opin Lipidol (2020) 31(1):20–6. doi: 10.1097/MOL.0000000000000653 PMC7413299 31789670

[72] Bains Y Erkin-Cakmak A Caccavello R Mulligan K Noworolski S Schwarz J-M. Isocaloric fructose restriction improves postprandial chylomicron and VLDL excursions in adolescents with obesity by reducing angiopoietin-like protein 3 and apolipoprotein CIII. Circ (2020) 142(Suppl-3):A16511–1. doi: 10.1161/circ.142.suppl_3.16511

[73] Krauss RM King SM. Remnant lipoprotein particles and cardiovascular disease risk. Best Pract Res Clin Endocrinol Metab (2023) 37(3):101682. doi: 10.1016/j.beem.2022.101682 35718703

[74] Wen Y Chen YQ Konrad RJ. The regulation of triacylglycerol metabolism and lipoprotein lipase activity. Adv Biol (2022) 6(10):e2200093. doi: 10.1002/adbi.202200093 35676229

[75] Wu SA Kersten S Qi L. Lipoprotein lipase and its regulators: an unfolding story. Trends Endocrinol Metab (2021) 32(1):48–61. doi: 10.1016/j.tem.2020.11.005 33277156 PMC8627828

[76] Kumari A Kristensen KK Ploug M Winther AML. The importance of lipoprotein lipase regulation in atherosclerosis. Biomedicines (2021) 9(7):782. doi: 10.3390/biomedicines9070782 34356847 PMC8301479

[77] He PP Jiang T OuYang XP Liang YQ Zou JQ Wang Y. Lipoprotein lipase: Biosynthesis, regulatory factors, and its role in atherosclerosis and other diseases. Clinica Chimica Acta (2018) 480:126–37. doi: 10.1016/j.cca.2018.02.006 29453968

[78] Li J Li L Guo DM Li SY Zeng YX Liu CH. Triglyceride metabolism and angiopoietin-like proteins in lipoprotein lipase regulation. Clinica Chimica Acta (2020) 503:19–34. doi: 10.1016/j.cca.2019.12.029 31923423

[79] Valladolid-Acebes I Berggren PO Juntti-Berggren L. Apolipoprotein ciii is an important piece in the type-1 diabetes jigsaw puzzle. Int J Mol Sci (2021) 22(2):1–13. doi: 10.3390/ijms22020932 PMC7832341 33477763

[80] de la Parra Soto LG Gutiérrez-Uribe JA Sharma A Ramírez-Jiménez AK. Is Apo-CIII the new cardiovascular target? An analysis of its current clinical and dietetic therapies. Nutrition Metab Cardiovasc Diseases (2022) 32(2):295–308. doi: 10.1016/j.numecd.2021.09.035 34895805

[81] Dib I Khalil A Chouaib R El-Makhour Y Noureddine H. Apolipoprotein C-III and cardiovascular diseases: when genetics meet molecular pathologies. Mol Biol Rep (2021) 48(1):875–86. doi: 10.1007/s11033-020-06071-5 PMC7778846 33389539

[82] Aguilar-Recarte D Palomer X Vázquez-Carrera M. Uncovering the role of apolipoprotein C-III in insulin resistance. Clinica e Investigacion en Arteriosclerosis (2021) 33(2):108–15. doi: 10.1016/j.arteri.2020.09.003 33303217

[83] Chen YQ Zhen EY Russell AM Ehsani M Siegel RW Qian Y. Decoding the role of angiopoietin-like protein 4/8 complex-mediated plasmin generation in the regulation of LPL activity. J Lipid Res (2023) 64(10):100441. doi: 10.1016/j.jlr.2023.100441 37666362 PMC10550811

[84] Wolska A Lo L Sviridov DO Pourmousa M Pryor M Ghosh SS. A dual apolipoprotein C-II mimetic-apolipoprotein C-III antagonist peptide lowers plasma triglycerides. Sci Transl Med (2020) 12(528):eeaaw7905. doi: 10.1126/scitranslmed.aaw7905 PMC8359806 31996466

[85] Huynh K. Dual apoC-II mimetic and apoC-III antagonist for hypertriglyceridaemia. Nat Rev Cardiol (2020) 17(4):201. doi: 10.1038/s41569-020-0351-6 32055035

[86] Vergès B Adiels M Boren J Barrett PH Watts GF Chan D. Interrelationships between the kinetics of VLDL subspecies and hdl catabolism in abdominal obesity: A multicenter tracer kinetic study. J Clin Endocrinol Metab (2014) 99(11):4281–90. doi: 10.1210/jc.2014-2365 25077901

[87] Endo Y Fujita M Ikewaki K. HDL functions—Current status and future perspectives. Biomolecules (2023) 13(1):105. doi: 10.3390/biom13010105 36671490 PMC9855960

[88] Vitali C Khetarpal SA Rader DJ. HDL cholesterol metabolism and the risk of CHD: new insights from human genetics. Curr Cardiol Rep (2017) 19(12):133. doi: 10.1007/s11886-017-0940-0 29103089

[89] Kontush A. HDL. and reverse remnant-cholesterol transport (RRT): relevance to cardiovascular disease. Trends Mol Med (2020) 26(12):1086–100. doi: 10.101/jmolmed.2020.07.005 32861590

[90] Kontush A Martin M Brites F. Sweet swell of burning fat: emerging role of high-density lipoprotein in energy homeostasis. Curr Opin Lipidol (2023) 34(6):235–42. doi: 10.1097/MOL.0000000000000904 37797204

[91] Norwitz NG Soto-Mota A Kaplan B Ludwig DS Budoff M Kontush A. The lipid energy model: reimagining lipoprotein function in the context of carbohydrate-restricted diets. Metabolites (2022) 12(5):460. doi: 10.3390/metabo12050460 35629964 PMC9147253

[92] Tramontano D Bini S D’Erasmo L Arca M. Recent apolipoprotein CIII trials. Curr Opin Lipidol (2022) 33(6):309–18. doi: 10.1097/MOL.0000000000000849 36206093

[93] Ondrušková N Honzík T Kytnarová J Matoulek M Zeman J Hansíková H. Isoelectric focusing of serum apolipoprotein C-III as a sensitive screening method for the detection of O-glycosylation disturbances. Prague Med Rep (2015) 116(2):73–86. doi: 10.14712/23362936.2015.48 26093664

[94] Ginsberg HN Packard CJ Chapman MJ Borén J Aguilar-Salinas CA Averna M. Triglyceride-rich lipoproteins and their remnants: Metabolic insights, role in atherosclerotic cardiovascular disease, and emerging therapeutic strategies-a consensus statement from the European Atherosclerosis Society. Eur Heart J (2021) 42(47):4791–806. doi: 10.1093/eurheartj/ehab551 PMC8670783 34472586

[95] Chapman MJ Ginsberg HN. Evolocumab treatment of hypercholesterolemia in OSLER-1: enduring efficacy, tolerability, and safety over 5 years. J Am Coll Cardiol (2019) 74(17):2147–9. doi: 10.1016/j.jacc.2019.07.087 31648706

[96] Rodríguez-Mortera R Caccavello R Garay-Sevilla ME Gugliucci A. Higher ANGPTL3, apoC-III, and apoB48 dyslipidemia, and lower lipoprotein lipase concentrations are associated with dysfunctional visceral fat in adolescents with obesity. Clinica Chimica Acta (2020) 508:61–8. doi: 10.1016/j.cca.2020.05.014 32407781

[97] Paola Gutiérrez Castro K Patricia González A Caccavello R Garay-Sevilla ME Gugliucci A. Lean adolescents with insulin resistance display higher angiopoietin like protein 3, ApoC-III and chylomicron remnant dyslipidemia. Clin Chim Acta (2022) 526:43–8. doi: 10.1016/j.cca.2021.12.016 34971570

[98] Lustig RH Mulligan K Noworolski SM Tai VW Wen MJ Erkin-Cakmak A. Isocaloric fructose restriction and metabolic improvement in children with obesity and metabolic syndrome. Obesity (2016) 24(2):453–60. doi: 10.1002/oby.21371 PMC4736733 26499447

[99] Gugliucci A Lustig RH Caccavello R Erkin-Cakmak A Noworolski SM Tai VW. Short-term isocaloric fructose restriction lowers apoC-III levels and yields less atherogenic lipoprotein profiles in children with obesity and metabolic syndrome. Atherosclerosis (2016) 253:171–7. doi: 10.1016/j.atherosclerosis.2016.06.048 27451002

[100] May-Zhang L Liu M Black D Tso P. Apolipoprotein A5, a unique modulator of fasting and postprandial triglycerides. Biochim Biophys Acta Mol Cell Biol Lipids (2022) 1867(9):159185. doi: 10.1016/j.bbalip.2022.159185 35644522

[101] DiDonna NM Chen YQ Konrad RJ. Angiopoietin-like proteins and postprandial partitioning of fatty acids. Curr Opin Lipidol (2022) 33(1):39–46. doi: 10.1097/MOL.0000000000000798 34789669

[102] Masuda D Yamashita S. Postprandial hyperlipidemia and remnant lipoproteins. J Atheroscler Thromb (2017) 24(2):95–109. doi: 10.5551/jat.RV16003 27829582 PMC5305681

[103] Dallinga-Thie GM Kroon J Borén J Chapman MJ. Triglyceride-rich lipoproteins and remnants: targets for therapy? Curr Cardiol Rep (2016) 18(7):67. doi: 10.1007/s11886-016-0745-6 27216847 PMC4877422

[104] Sascău R Clement A Radu R Prisacariu C Stătescu C. Triglyceride-rich lipoproteins and their remnants as silent promoters of atherosclerotic cardiovascular disease and other metabolic disorders: A review. Nutrients (2021) 13(6):1774. doi: 10.3390/nu13061774 34067469 PMC8224751

[105] Krauss RM. Small dense low-density lipoprotein particles: clinically relevant? Curr Opin Lipidol (2022) 33(3):160–6. doi: 10.1097/MOL.0000000000000824 PMC9197986 35276699

[106] Tybjærg-Hansen A Nordestgaard BG Christoffersen M. Triglyceride-rich remnant lipoproteins are more atherogenic than LDL per particle: is this important? Eur Heart J (2023) 44(39):4196–8. doi: 10.1093/eurheartj/ehad419 37403539

[107] Wadström BN Wulff AB Pedersen KM Nordestgaard BG. Do triglyceride-rich lipoproteins equal low-density lipoproteins in risk of ASCVD? Curr Atheroscler Rep (2023) 25(1):795–803. doi: 10.1007/s11883-023-01153-8 37768410

[108] Johansen MØ Afzal S Vedel-Krogh S Nielsen SF Smith GD Nordestgaard BG. From plasma triglycerides to triglyceride metabolism: effects on mortality in the Copenhagen General Population Study. Eur Heart J (2023) 44(39):4174–82. doi: 10.1093/eurheartj/ehad330 37575001

[109] Botham KM Wheeler-Jones CPD. Postprandial lipoproteins and the molecular regulation of vascular homeostasis. Prog Lipid Res (2013) 52(4):446–64. doi: 10.1016/j.plipres.2013.06.001 23774609

[110] Botham K Bravo E Elliott J Wheeler-Jones C. Direct interaction of dietary lipids carried in chylomicron remnants with cells of the artery wall: implications for atherosclerosis development. Curr Pharm Des (2005) 11(28):3681–95. doi: 10.2174/138161205774580732 16305504

[111] Öörni K Lehti S Sjövall P Kovanen PT. Triglyceride-rich lipoproteins as a source of proinflammatory lipids in the arterial wall. Curr Med Chem (2018) 26(9):1701–10. doi: 10.2774/0929867325666180530094819 29848270

[112] Dalla-Riva J Garonna E Elliott J Botham KM Wheeler-Jones CP. Endothelial cells as targets for chylomicron remnants. Atheroscler Suppl (2010) 11(1):31–7. doi: 10.1016/j.atherosclerosissup.2010.04.001 20439166

[113] Sylvers-Davie KL Davies BSJ. Regulation of lipoprotein metabolism by ANGPTL3, ANGPTL4, and ANGPTL8. Am J Physiol Endocrinol Metab (2021) 321(4):E493–508. doi: 10.1152/ajpendo.00195.2021 PMC8560382 34338039

[114] Zhang R. The ANGPTL3-4-8 model, a molecular mechanism for triglyceride trafficking. Open Biol (2016) 6(4):150272. doi: 10.1098/rsob.150272 27053679 PMC4852456

[115] Zhang R Zhang K. An updated ANGPTL3-4-8 model as a mechanism of triglyceride partitioning between fat and oxidative tissues. Prog Lipid Res (2022) 85:101140. doi: 10.1016/j.plipres.2021.101140 34793860 PMC8760165

[116] Chen YQ Pottanat TG Siegel RW Ehsani M Qian YW Zhen EY. Angiopoietin-like protein 8 differentially regulates ANGPTL3 and ANGPTL4 during postprandial partitioning of fatty acids. J Lipid Res (2020) 61(8):1203–20. doi: 10.1194/jlr.RA120000781 PMC7397750 32487544

[117] Kristensen KK Leth-Espensen KZ Kumari A Grønnemose AL Lund-Winther AM Young SG. GPIHBP1 and ANGPTL4 utilize protein disorder to orchestrate order in plasma triglyceride metabolism and regulate compartmentalization of LPL activity. Front Cell Dev Biol (2021) 9:702508. doi: 10.3389/fcell.2021.702508 34336854 PMC8319833

[118] Ginsberg HN Goldberg IJ. Broadening the scope of dyslipidemia therapy by targeting APOC3 (Apolipoprotein C3) and ANGPTL3 (Angiopoietin-like protein 3). Arterioscler Thromb Vasc Biol (2023) 43(3):388–98. doi: 10.1161/ATVBAHA.122.317966 PMC9975058 36579649

[119] Kersten S. Role and mechanism of the action of angiopoietin-like protein ANGPTL4 in plasma lipid metabolism. J Lipid Res (2021) 62:100150. doi: 10.1016/j.jlr.2021.100150 34801488 PMC8666355

[120] Aryal B Price NL Suarez Y Fernández-Hernando C. ANGPTL4 in metabolic and cardiovascular disease. Trends Mol Med (2019) 25(8):723–34. doi: 10.1016/j.molmed.2019.05.010 PMC6779329 31235370

[121] Abu-Farha M Cherian P Qaddoumi MG AlKhairi I Sriraman D Alanbaei M. Increased plasma and adipose tissue levels of ANGPTL8/Betatrophin and ANGPTL4 in people with hypertension. Lipids Health Dis (2018) 17(1):35. doi: 10.1186/s12944-018-0681-0 29490644 PMC5831738

[122] Guo C Wang C Deng X He J Yang L Yuan G. ANGPTL8 in metabolic homeostasis: More friend than foe? Open Biol (2021) 11(9):210106. doi: 10.1098/rsob.210106 34582711 PMC8478524

[123] Geladari E Tsamadia P Vallianou NG. ANGPTL3 Inhibitors: Their role in cardiovascular disease through regulation of lipid metabolism. Circ J (2019) 83(2):267–73. doi: 10.1253/circj.CJ-18-0442 30504621

[124] Shang R Rodrigues B. Lipoprotein lipase and its delivery of fatty acids to the heart. Biomolecules (2021) 11(7):1016. doi: 10.3390/biom11071016 34356640 PMC8301904

[125] Sachs S Götz A Finan B Feuchtinger A DiMarchi RD Döring Y. GIP receptor agonism improves dyslipidemia and atherosclerosis independently of body weight loss in preclinical mouse model for cardio-metabolic disease. Cardiovasc Diabetol (2023) 22(1):217. doi: 10.1186/s12933-023-01940-2 37592302 PMC10436634

[126] Kanoski SE Ong ZY Fortin SM Schlessinger ES Grill HJ. Liraglutide, leptin and their combined effects on feeding: Additive intake reduction through common intracellular signalling mechanisms. Diabetes Obes Metab (2015) 17(3):285–93. doi: 10.1111/dom.12423 PMC4320650 25475828

[127] Yang Y Choi PP Smith WW Xu W Ma D Cordner ZA. Exendin-4 reduces food intake via the PI3K/AKT signaling pathway in the hypothalamus. Sci Rep (2017) 7(1):6936. doi: 10.1038/s41598-017-06951-0 28761132 PMC5537284

[128] Williams DL Baskin DG Schwartz MW. Evidence that intestinal glucagon-like peptide-1 plays a physiological role in satiety. Endocrinology (2009) 150(4):1680–7. doi: 10.1210/en.2008-1045 PMC2659282 19074583

[129] Mukherjee K Wang R Xiao C. Release of lipids stored in the intestine by glucagon-like peptide-2 involves a gut-brain neural pathway. Arterioscler Thromb Vasc Biol (2023) 44(1):192–201. doi: 10.1161/ATVBAHA.123.320032 37970717

[130] Nahmias A Stahel P Tian L Xiao C Lewis GF. GLP-1 (Glucagon-like peptide-1) is physiologically relevant for chylomicron secretion beyond its known pharmacological role. Arterioscler Thromb Vasc Biol (2021) 41(6):1893–900. doi: 10.1161/ATVBAHA.121.316311 33951941

[131] Taskinen MR Matikainen N Björnson E Söderlund S Ainola M Hakkarainen A. Role of endogenous incretins in the regulation of postprandial lipoprotein metabolism. Eur J Endocrinol (2022) 187(1):75–84. doi: 10.1530/EJE-21-1187 35521766

[132] Stahel P Xiao C Nahmias A Lewis GF. Role of the gut in diabetic dyslipidemia. Front Endocrinol (Lausanne) (2020) 11:116. doi: 10.3389/fendo.2020.00116 32231641 PMC7083132

[133] Leohr J Kjellsson MC. Evaluation of postprandial total triglycerides within the TIGG model for characterizing postprandial response of glucose, insulin, and GLP-1. CPT Pharmacometrics Syst Pharmacol (2023) 12(10):1539–1540. doi: 10.1002/psp4.13030 PMC10583241 37667531

[134] Stahel P Xiao C Nahmias A Tian L Lewis GF. Multi-organ coordination of lipoprotein secretion by hormones, nutrients and neural networks. Endocr Rev (2021) 42(6):815–38. doi: 10.1210/endrev/bnab008 PMC8599201 33743013

[135] Ma T Lu W Wang Y Qian P Tian H Gao X. An oral GLP-1 and GIP dual receptor agonist improves metabolic disorders in high fat-fed mice. Eur J Pharmacol (2022) 914:174635. doi: 10.1016/j.ejphar.2021.174635 34800466

[136] Taskinen MR Björnson E Matikainen N Söderlund S Pietiläinen KH Ainola M. Effects of liraglutide on the metabolism of triglyceride-rich lipoproteins in type 2 diabetes. Diabetes Obes Metab (2021) 23(5):1191–201. doi: 10.1111/dom.14328 33502078

[137] Borén J Taskinen MR Olofsson SO Levin M. Ectopic lipid storage and insulin resistance: A harmful relationship. J Intern Med (2013) 274(1):25–40. doi: 10.1111/joim.12071 23551521

[138] González AP Flores-Ramírez A Gutiérrez-Castro KP Luévano-Contreras C Gómez-Ojeda A Sosa-Bustamante GP. Reduction of small dense LDL and Il-6 after intervention with Plantago psyllium in adolescents with obesity: a parallel, double blind, randomized clinical trial. Eur J Pediatr (2021) 180(8):2493–503. doi: 10.1007/s00431-021-04064-5 33861390

[139] Chye Ooi T G Nordestgaard B. Methods to study postprandial lipemia. Curr Vasc Pharmacol (2011) 9(3):302–8. doi: 10.2174/157016111795495567 21314627

[140] Kolovou G D Mikhailidis D P Kovar J Lairon D G. Nordestgaard B Chye Ooi T. Assessment and clinical relevance of non-fasting and postprandial triglycerides: an expert panel statement. Curr Vasc Pharmacol (2011) 9(3):258–70. doi: 10.2174/157016111795495549 21314632

[141] Yanai H Adachi H Hakoshima M Katsuyama H. Postprandial hyperlipidemia: its pathophysiology, diagnosis, atherogenesis, and treatments. Int J Mol Sci (2023) 24(18):13942. doi: 10.3390/ijms241813942 37762244 PMC10530470

[142] Kolovou GD Watts GF Mikhailidis DP Pérez-Martínez P Mora S Bilianou H. Postprandial hypertriglyceridaemia revisited in the era of non-fasting lipid profile testing: A 2019 expert panel statement, main text. Curr Vasc Pharmacol (2019) 17(5):498–514. doi: 10.2174/1570161117666190507110519 31060488

[143] Yoshinaga MY Quintanilha BJ Chaves-Filho AB Miyamoto S Sampaio GR Rogero MM. Postprandial plasma lipidome responses to a high-fat meal among healthy women. J Nutr Biochem (2021) 97. doi: 10.1016/j.jnutbio.2021.108809 34192591

[144] Metelcová T Hainer V Hill M Kalousková P Vrbíková J Šrámková P. Postprandial triglyceride, glucose and insulin levels 10 years after bariatric surgery in women with severe obesity - A pilot study: part 2 - biliopancreatic diversion. Physiol Res (2023) 72(S4):S405–10. doi: 10.33549/physiolres.935179 PMC10830165 38116774

[145] Langsted A Nordestgaard BG. Nonfasting versus fasting lipid profile for cardiovascular risk prediction. Pathology (2019) 51(2):131–41. doi: 10.1016/j.pathol.2018.09.062 30522787

[146] Mihas C D Kolovou G Mikhailidis D P Kovar J Lairon D Nordestgaard B G. Diagnostic value of postprandial triglyceride testing in healthy subjects: A meta-analysis. Curr Vasc Pharmacol (2011) 999(999):1–20. doi: 10.2174/1570211213146311611 21314631

[147] Hanada H Mugii S Okubo M Maeda I Kuwayama K Hidaka Y. Establishment of chemiluminescence enzyme immunoassay for apolipoprotein B-48 and its clinical applications for evaluation of impaired chylomicron remnant metabolism. Clin Chim Acta (2012) 413(1–2):160–5. doi: 10.1016/j.cca.2011.09.013 21958700

[148] Sakai N Uchida Y Ohashi K Hibuse T Saika Y Tomari Y. Measurement of fasting serum apoB-48 levels in normolipidemic and hyperlipidemic subjects by ELISA. J Lipid Res (2003) 44(6):1256–62. doi: 10.1194/jlr.M300090-JLR200 12671037

[149] Ganda OP. Triglyceride-rich lipoproteins, remnant-cholesterol, and atherosclerotic cardiovascular disease. Curr Opin Lipidol (2023) 34(3):105–13. doi: 10.1097/MOL.0000000000000875 36924359

[150] Turner SM Murphy EJ Neese RA Antelo F Thomas T Agarwal A. Measurement of TG synthesis and turnover in vivo by 2H 2O incorporation into the glycerol moiety and application of MIDA. Am J Physiol Endocrinol Metab (2003) 285(4 48-4):E790–E803. doi: 10.1152/ajpendo.00402.2002 12824084

[151] Lim JS Mietus-Snyder M Valente A Schwarz JM Lustig RH. The role of fructose in the pathogenesis of NAFLD and the metabolic syndrome. Nat Rev Gastroenterol Hepatol (2010) 7(5):251–64. doi: 10.1038/nrgastro.2010.41 20368739

[152] Reith C Armitage J. Management of residual risk after statin therapy. Atherosclerosis (2016) 245:161–70. doi: 10.1016/j.atherosclerosis.2015.12.018 26722833

[153] Mszar R Bart S Sakers A Soffer D Karalis DG. Current and emerging therapies for atherosclerotic cardiovascular disease risk reduction in hypertriglyceridemia. J Clin Med (2023) 12(4):1382. doi: 10.3390/jcm12041382 36835917 PMC9962307

[154] Keech A Simes R Barter P Best J Scott R Taskinen MR. Effects of long-term fenofibrate therapy on cardiovascular events in 9795 people with type 2 diabetes mellitus (the FIELD study): randomised controlled trial. Lancet (2005) 366(9500):1849–61. doi: 10.1016/S0140-6736(05)67667-2 16310551

[155] Sirtori CR. The FIELD study. Lancet (2006) 367(9517):1141–2. doi: 10.1016/S0140-6736(06)68500-0 16616551

[156] McQueen M. Long term fenofibrate did not reduce major coronary events but may reduce total CVD events in type 2 diabetes. Evid Based Med (2006) 11(3):86. doi: 10.1136/ebm.11.3.86 17213107

[157] Hirano T Ito Y. The influence of triglycerides on small dense low-density lipoprotein cholesterol levels is attenuated in low low-density lipoprotein-cholesterol range: Implications for the negative results of the PROMINENT trial. J Diabetes Investig (2023) 14(7):902–6. doi: 10.1111/jdi.14013 PMC10286789 37016542

[158] Hirano T. No change in small low-density lipoprotein cholesterol levels with pemafibrate might explain the negative results of the PROMINENT trial. J Diabetes Investig (2023) 14(4):630–1. doi: 10.1111/jdi.13983 PMC10034948 36708089

[159] Chukwurah MI Miller M. Fibrates, hypertriglyceridemia, and CVD risk: where do we stand after the PROMINENT trial for triglyceride lowering? Curr Cardiol Rep (2023) 25(9):987–92. doi: 10.1007/s11886-023-01926-2 37505399

[160] Miller M. Icosapent ethyl for hypertriglyceridemia: Insights from the REDUCE-IT Trial. Future Cardiol (2019) 15(6):391–4. doi: 10.2217/fca-2019-0054 31524535

[161] Doi T Langsted A Nordestgaard BG. A possible explanation for the contrasting results of REDUCE-IT vs. STRENGTH: Cohort study mimicking trial designs. Eur Heart J (2021) 42(47):4807–17. doi: 10.193/eurheartj.ehab555 34455435

[162] Picard F Bhatt DL Ducrocq G Ohman EM Goto S Eagle KA. Generalizability of the REDUCE-IT trial and cardiovascular outcomes associated with hypertriglyceridemia among patients potentially eligible for icosapent ethyl therapy: An analysis of the REduction of Atherothrombosis for Continued Health (REACH) registry. Int J Cardiol (2021) 340:96–104. doi: 10.1016/j.ijcard.2021.08.031 34450192

[163] Calcaterra I Lupoli R Di Minno A Di Minno MND. Volanesorsen to treat severe hypertriglyceridaemia: A pooled analysis of randomized controlled trials. Eur J Clin Invest (2022) 52(11):e13841. doi: 10.1111/eci.13841 35851450 PMC9788245

[164] Esan O Wierzbicki AS. Volanesorsen in the treatment of familial chylomicronemia syndrome or hypertriglyceridaemia: Design, development and place in therapy. Drug Des Devel Ther (2020) 14:2623–36. doi: 10.2147/DDDT.S224771 PMC7351689 32753844

[165] D’Erasmo L Gallo A Di Costanzo A Bruckert E Arca M. Evaluation of efficacy and safety of antisense inhibition of apolipoprotein C-III with volanesorsen in patients with severe hypertriglyceridemia. Expert Opin Pharmacother (2020) 21(4):1675–84. doi: 10.1080/14656566.2020.1787380 32646313

[166] Gouni-Berthold I Schwarz J Berthold HK. Updates in drug treatment of severe hypertriglyceridemia. Curr Atheroscler Rep (2023) 25(10):701–9. doi: 10.1007/s11883-023-01140-z PMC10564803 37642858

[167] Prohaska TA Alexander VJ Karwatowska-Prokopczuk E Tami J Xia S Witztum JL. APOC3 inhibition with volanesorsen reduces hepatic steatosis in patients with severe hypertriglyceridemia. J Clin Lipidol (2023) 17(3):406–11. doi: 10.1016/j.jacl.2023.04.007 37164837

[168] Larouche M Khoury E Brisson D Gaudet D. Inhibition of angiopoietin-like protein 3 or 3/8 complex and apoC-III in severe hypertriglyceridemia. Curr Atheroscler Rep (2023) 25(11):1101–11. doi: 10.1007/s11883-023-01179-y 38095804

[169] Witztum JL Gaudet D Arca M Jones A Soran H Gouni-Berthold I. Volanesorsen and triglyceride levels in familial chylomicronemia syndrome: Long-term efficacy and safety data from patients in an open-label extension trial. J Clin Lipidol (2023) 17(3):342–55. doi: 10.1016/j.jacl.2023.03.007 37100699

[170] Okazaki H Gotoda T Ogura M Ishibashi S Inagaki K Daida H. Current diagnosis and management of primary chylomicronemia. J Atheroscler Thromb (2021) 28(9):883–904. doi: 10.5551/jat.RV17054 33980761 PMC8532063

[171] Chan DC Ying Q Watts GF. Recent dynamic studies of the metabolism of atherogenic lipoproteins: elucidating the mode of action of new therapies. Curr Opin Lipidol (2021) 32(6):378–85. doi: 10.1097/MOL.0000000000000795 34636776

